# Self-Assembly of Linear Three-Ring Aromatic Thiols on Au(111)

**DOI:** 10.1021/acs.jpclett.5c03949

**Published:** 2026-02-02

**Authors:** Verena Müller, Anna-Laurine Gaus, Daniel Hüger, Julian Picker, Christof Neumann, Max von Delius, Andrey Turchanin

**Affiliations:** † Institute of Physical Chemistry, 9378 Friedrich Schiller University Jena, 07743 Jena, Germany; ‡ Institute of Organic Chemistry, University of Ulm, 89081 Ulm, Germany

## Abstract

We report on the self-assembly of linear three-ring aromatic thiols on Au(111)/mica substrates. Our study examines terphenylthiol (TPT) derivatives with distinct terminal groups −F (FTPT), −CF_3_ (CF_3_TPT) and −NO_2_ (NTPT) as well as a pyridinebiphenyl (PyBPT) compound. Using complementary surface science techniques—X-ray photoelectron spectroscopy (XPS), low-energy electron diffraction (LEED), and scanning tunneling microscopy (STM)—we elucidate the structural properties of the resulting self-assembled monolayers (SAMs). The TPT, FTPT, CF_3_TPT and PyBPT molecules form densely packed SAMs with hexagonal unit cells exhibiting an area of 21.55 Å^2^ per molecule. For the NTPT SAM, two different molecular arrangements were observed to coexist: a hexagonal structure and a squared structure, with areas per molecule of 21.55 and 42.25 Å^2^, respectively.

## Graphical abstract

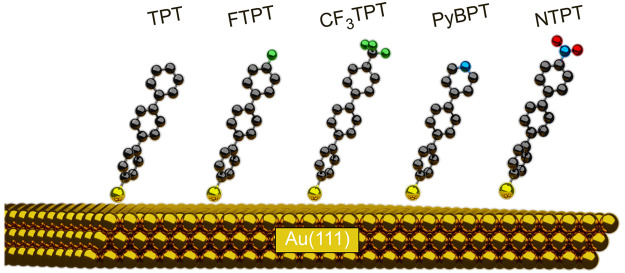

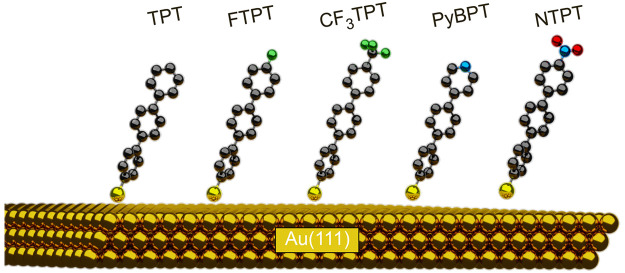

Thiol-based self-assembled monolayers (SAMs) on metal substrates^1,2^ play an important role in molecular nanotechnology, as they enable precise tailoring of interfacial material properties for both applied and fundamental research, including molecular electronics,^3^ catalysis,^4^ biosensing,^5^ and studies of interfacial phenomena.^6^ By designing the molecular backbones and terminal functional groups of the SAM forming molecules, it is possible to obtain control over the structure at the nanoscale, stability, and chemical reactivity of the resulting monolayers, as well as over their response to irradiation with light or charged particles (see, *e.g*., refs 7–10 ). Aromatic SAMs are of particular interest because irradiation with low-energy electrons can induce intermolecular cross-linking, converting the SAM into a molecular two-dimensional (2D) material known as a carbon nanomembrane (CNM).^8,11^ The chemical and physical properties of CNMs depend strongly on the choice of the SAM precursor molecules,^12^ providing a versatile platform for the generation of 2D materials for applications in nanolithography,^13,14^ nanofiltration,^15–19^ molecular interferometry,^20^ biochips,^21,22^ ultrasensitive biosensors and actuators,^23–25^ photoactive components in field-effect transistors,^26–28^ and energy-conversion devices,^29,30^ among others.

A distinctive property of CNMs is the presence of high-density subnanometer pores,^16^ which governs the permeation of gases^15–19^ as well as both liquid and solid electrolytes.^17,31–34^ For example, CNMs synthesized from 1,1′,4′,1″-terphenyl-4-thiol (TPT) SAMs on gold substrates combine excellent mechanical stability^35,36^ with remarkable selectivity for the permeation of water vapor and various gases (*e.g*., He, Ne, D_2_, CO_2_, Ar, O_2_)^16,18^ as well as can regulate permeation of protons and lithium ions in electrochemical devices.^32–34^ These characteristics make TPT-derived CNMs highly attractive for gas dehydration, hydrogen separation, decarbonization and energy storage technologies.

Motivated by the results reported for TPT SAMs, we present a systematic study of the formation of SAMs on gold substrates from various linear aromatic thiols, consisting similar as TPT of three aromatic rings, as potential precursors for CNM synthesis. Employing TPT SAM as a reference system, we investigate SAMs formed from 4″-fluoro-[1,1′:4′,1″-terphenyl]-4-thiol (FTPT), 4″-(trifluoromethyl)-[1,1′:4′,1″-terphenyl]-4-thiol (CF_3_TPT), 4′-(pyridine-4-yl)-[1,1′-biphenyl]-4-thiol (PyBPT), and 4″-nitro-[1,1′:4′,1″-terphenyl]-4-thiol (NTPT). Although some spectroscopic studies of FTPT, CF_3_TPT, and NTPT SAMs on gold have already been reported in the literature,^37,38^ their microscopic characterization has remained unavailable. Here, SAMs of all five compounds were prepared from solution on Au/mica substrates with the preferential (111) orientation and analyzed in a comparative study using complementary surface science techniques, including X-ray photoelectron spectroscopy (XPS), scanning tunneling microscopy (STM), and low-energy electron diffraction (LEED). The results presented below enable us to establish correlations between constitution of the SAM forming molecules and structural properties of the resulting monolayers.

The modified terphenyl compounds were prepared *via* customized multistep syntheses to accommodate the differing reactivity of the individual functional head groups (Figure 1; see the Supporting Information for details). For the PyBPT derivative, the goal was to replace the benzene unit of commercially available TPT with a more polar, electron-deficient pyridine ring. To this end, 4-(4′,-bromophenyl)­pyridine was first prepared *via* Suzuki coupling, followed by a second cross-coupling reaction to introduce a para-methyl-protected thiol substituent. Subsequent thiolate exchange afforded the *tert*-butyl-protected thiol, which was cleaved to the free thiol under acidic conditions (see Figure S1). For the synthesis of the fluorinated derivatives FTPT and CF_3_TPT, the thiol functionality was introduced to 4′-bromo-3-iodo-1,1′-biphenyl and concurrently protected using 2-ethylhexyl-3-mercaptopropionate, following the procedure of Itoh and Mase.^39^ Subsequent coupling with 4-fluorophenyl- or 4-(trifluoromethyl)­phenylboronic acid afforded the respective precursors of the derivatives. Deprotection under basic conditions with sodium ethoxide and subsequent acidic workup yielded the corresponding free thiols.

**Figure 1:**
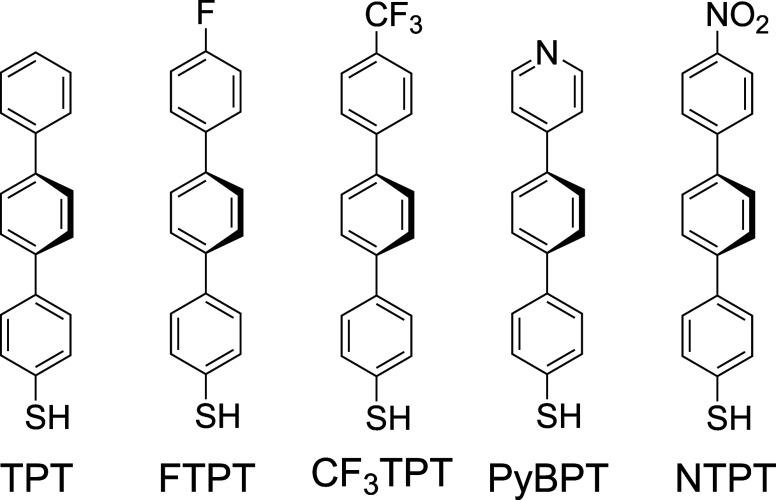
Molecular structures of the precursor molecules: 1,1′,4′,1″-terphenyl-4-thiol (TPT), 4″-fluoro-[1,1′’:4′,1″-terphenyl]-4-thiol (FTPT), 4″-(trifluoromethyl)-[1,1′:4′,1″-terphenyl]-4-thiol (CF_3_TPT), 4′-(pyridin-4-yl)-[1,1′-biphenyl]-4-thiol (PyBPT), and 4″-nitro-[1,1′:4′,1″-terphenyl]-4-thiol (NTPT).

The nitro-substituted NTPT compound was synthesized following a reported procedure,^37^ in which the hydroxyl group of 4″-nitro-4-hydroxyterphenyl was first converted to a dimethylthiocarbamate. A Newman–Kwart rearrangement afforded the corresponding thiourethane, which was subsequently cleaved under basic conditions and, after acidic workup, provided the free thiol anchoring group. NTPT, FTPT and CF_3_TPT were purified by column chromatography and all compounds were characterized *via* nuclear magnetic resonance (NMR) spectroscopy and mass spectrometry (MS, see the Supporting Information).

Next, all five molecular compounds were used to form SAMs on Au/mica substrates with the typical (111) orientation of the surface (see the Materials). The obtained samples were then studied by XPS. Figure 2 shows high-resolution XP spectra of the characteristic S 2p, C 1s, N 1s, O 1s and F 1s signals. As seen from the S 2p spectra, for all samples a single doublet at the binding energies (BEs) of 161.9 ± 0.1 eV (S 2p_3/2_) and 163.1 ± 0.1 eV (S 2p_1/2_) is observed. This BE is characteristic of the formation of thiolate bonds on gold,^40^ suggesting a successful formation of the SAMs. In contrast, C 1s, N 1s, O 1s and F 1s spectra show distinct differences which result from variations in the chemical constitution of the respective TPT, FTPT, CF_3_TPT, PyBPT, and NTPT SAMs. In the following we analyze these spectra in detail.

**Figure 2:**
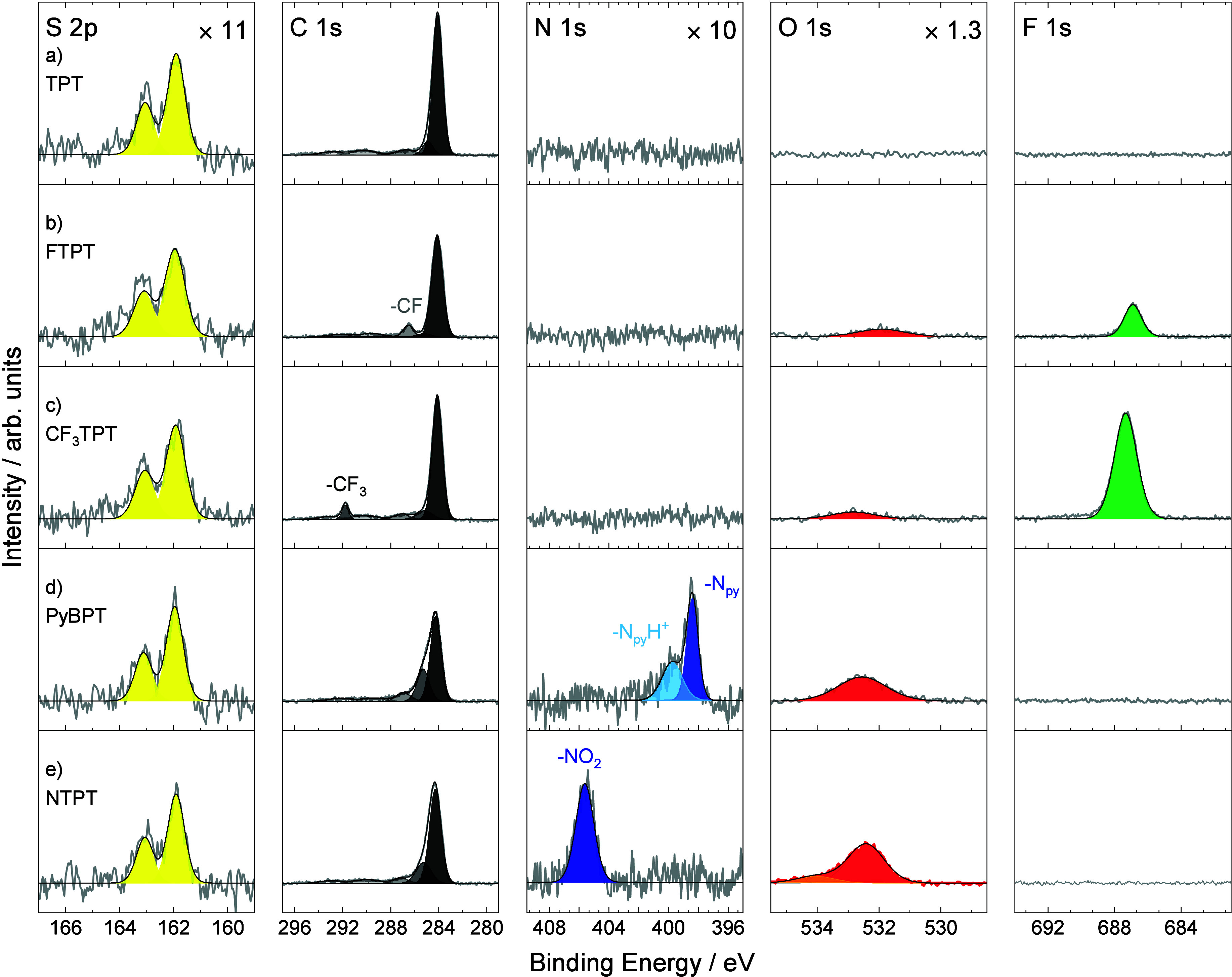
High-resolution S 2p, C 1s, N 1s, O 1s and F 1s XP spectra of the formed a) TPT, b) FTPT, c) CF_3_TPT, d) PyBPT, and e) NTPT SAMs on Au/mica. S 2p, N 1s, and O 1s spectra were multiplied with the given factors for better visualization.

The high-resolution C 1s XP spectrum of the TPT SAM consists of a narrow main peak at a BE of 284.1 eV with a full width at half-maximum (FWHM) of 1.0 eV (Figure 2a). This feature is attributed to C–C and C–H bonds of the molecules. This peak is accompanied by a shoulder at a BE of 284.9 eV assigned to C–S bonds. In addition, aromatic shake-ups are visible (Table S1). The effective thickness of the SAM is 13 ± 2 Å. The calculated C:S ratio obtained using Beer–Lambert law (see the Methods for details) is (18.5 ± 1.5):1 fitting well to the stoichiometry. Besides carbon and sulfur, no other elements were detected *via* XPS, confirming the high quality of the formed SAM. All these results match well to the previous study for TPT SAMs on Au and confirm the successful preparation.^12,41,42^

Next, FTPT SAM was investigated (Figure 2b). The high-resolution C 1s spectrum shows similar features compared to TPT having a main contribution according to C–C/C–H bonds at a BE of 284.1 eV with a shoulder assigned to C–S bonds at a BE of 285.2 eV. In contrast to TPT, the C–F group is visible as an additional narrow peak at a BE of 286.5 eV with a FWHM of 1.0 eV.^38^ Accordingly, a F 1s peak can be detected at a BE of 686.9 eV. Some minor traces of oxygen are visible in the spectrum. The effective thickness of the SAM is 11 ± 2 Å, which is slightly lower in comparison to the TPT SAM. The S:F:C ratios were determined to 1:(0.9 ± 0.1):(17.3 ± 1.5). The obtained results match well to the molecule structure and are consistent with earlier studies of F-TPT SAMs.^38^

Figure 2c shows the XPS data of CF_3_TPT SAMs. The C 1s spectrum has a main peak at a BE of 284.1 eV assigned to C–C/C–H bonds with a shoulder at a BE of 285.2 eV according to C–S bonds. This resembles the results of the TPT and FTPT SAMs having identical molecular backbones. The −CF_3_ group is clearly detected in the C 1s spectrum at the BE of 291.8 eV with a FWHM of 0.6 eV.^38^ The F 1s signal shows a single peak at a BE of 687.3 eV, which has clearly larger intensity than the F 1s peak of FTPT due to the higher number of fluorine atoms. A slightly higher BE of the F 1s peak of CF_3_TPT SAMs in comparison to the FTPT SAM agrees with a stronger electronegativity of the −CF_3_ group compared to the −F group. The calculated S:F:C ratio is of 1:(3.9 ± 0.5):(18.5 ± 1.5). Similarly to FTPT SAMs, only some minor traces of oxygen are present in the O 1s spectrum, confirming the high quality of the formed SAM. The calculated effective thickness of 13 ± 2 Å is similar as for TPT SAMs.

Figure 2d presents the XPS data for PyBPT SAMs. The C 1s spectrum shows characteristic differences in comparison to the previously discussed SAMs. The main peak assigned to C–C/C–H bonds at a BE of 284.3 eV has a lower intensity, whereas the intensity of the shoulder at a BE of 285.3 eV is increased, which results from the contribution of the C–N bonds of the pyridine group. The presence of the pyridine group is clearly manifested in a narrow peak in the N 1s spectrum with a characteristic BE of 398.4 eV and a FWHM of 0.9 eV.^43,44^ This main feature is accompanied by a broad shoulder at a BE of 399.8 eV (FWHM 1.6 eV), which is due to the protonated pyridines.^43,44^ The calculated effective thickness is 13 ± 2 Å. In the O 1s spectrum, a small peak at a BE of 532.5 eV (orange) was detected which may result from some airborne hydrocarbon or water adsorption on the SAM, as the pyridine groups can react with CO_2_ from atmosphere to form salt-like structures (N_py_H^+ −^OOC).^45^ This is further reflected in a slightly increased carbon content as seen from the calculated stoichiometric S:N:C ratio of 1:(1.1 ± 0.1):(19.7 ± 1.5).

Finally, in Figure 2e the XPS results for NTPT SAMs are presented. The C 1s spectrum consists of a main peak at a BE of 284.3 eV accompanied by a shoulder at a BE of 285.2 eV assigned to the C–S and C–N bond of the nitro group. The N 1s spectrum is represented by a single peak at a BE of 405.6 eV related to the −NO_2_ group.^37^ The observed XP spectra fit well those measured for 4′-nitrobiphenyl-4-thiol (NBPT) SAMs, which are structurally similar only consisting of one phenyl ring shorter derivative.^46^ The nitro group also contributes to the O 1s spectrum at a BE of 532.4 eV.^46^ A small shoulder at a BE of 534.1 eV may result from a slight amount of adsorbed water.^47^ The effective thickness of the NTPT SAMs is 13 ± 2 Å and the S:N:O:C ratios are 1:(1.1 ± 0.1):(2.3 ± 0.3):(18.6 ± 1.5), which fits well to the expected stoichiometry.

After the spectroscopic characterization of TPT, FTPT, CF_3_TPT, PyBPT, and NTPT SAMs, we studied their structure on Au(111) *via* LEED and STM (Figure 3). In Figure 3a results for the TPT SAM are presented. As seen in the molecular resolved STM image, this monolayer exhibits a highly ordered structure, which also extends over large sample areas (see Figure S36a). From the line profile analysis, the nearest neighbor distance of the TPT molecules was found to be 5.2 ± 1.0 Å (red line in Figure S36a). Notably, every second molecule along this line profile exhibits a different STM height. The line profile along the other lattice vector direction shows molecules with almost identical heights (blue line in Figure S36a). Based on these data, the unit cell of the TPT SAM can be identified as presented by a yellow line in Figure 3a and Figure S36a with the lattice parameters |*a⃗*_1_| = 10.3 ± 2.0 Å, |*a⃗*_2_| = 5.2 ± 1.0 Å, (*a⃗*_1_,*a⃗*_2_) = 120°, where |*a⃗*_1_| and |*a⃗*_1_| are the lattice vectors with their enclosing angle ∠(*a⃗*_1_,*a⃗*_2_). This unit cell consists of two TPT molecules and corresponds to a $2\sqrt{3}\times \sqrt{3}$ R30° superstructure with respect to the Au(111) surface as it was also reported in the literature.^12,48–51^ However, a careful analysis of Figure 3a shows the presence of additional structural features, which lead to a bigger unit cell highlighted with blue lines. Here, every fourth row of molecules along the lattice vector direction possesses a higher STM height (brighter contrast, blue line profile in Figure S36b). This unit cell has the lattice parameters of |*a⃗*_1_| = 10.3 ± 2.0 Å, |*a⃗*_2_| = 20.3 ± 4.0 Å, ∠(*a⃗*_1_,*a⃗*_2_) = 120° and consists of 8 TPT molecules and forms a higher order commensurate $2\sqrt{3}\times 4\sqrt{3}R30^{{}^{\circ}}$ superstructure. Note that this periodicity is interrupted in the central part of the STM image of Figure 3a by a dislocation defect. The larger $2\sqrt{3}\times 4\sqrt{3}R30^{{}^{\circ}}$ superstructure exhibits a periodicity along the red line profile (Figure S36b), with every second molecule differing in height, which strongly indicates that the TPT SAM comprises both the $2\sqrt{3}\times \sqrt{3}R30^{{}^{\circ}}$ and $2\sqrt{3}\times 4\sqrt{3}R30^{{}^{\circ}}$ superstructures. The fast Fourier transform (FFT) of the TPT SAM confirms the expected hexagonal structures.

**Figure 3:**
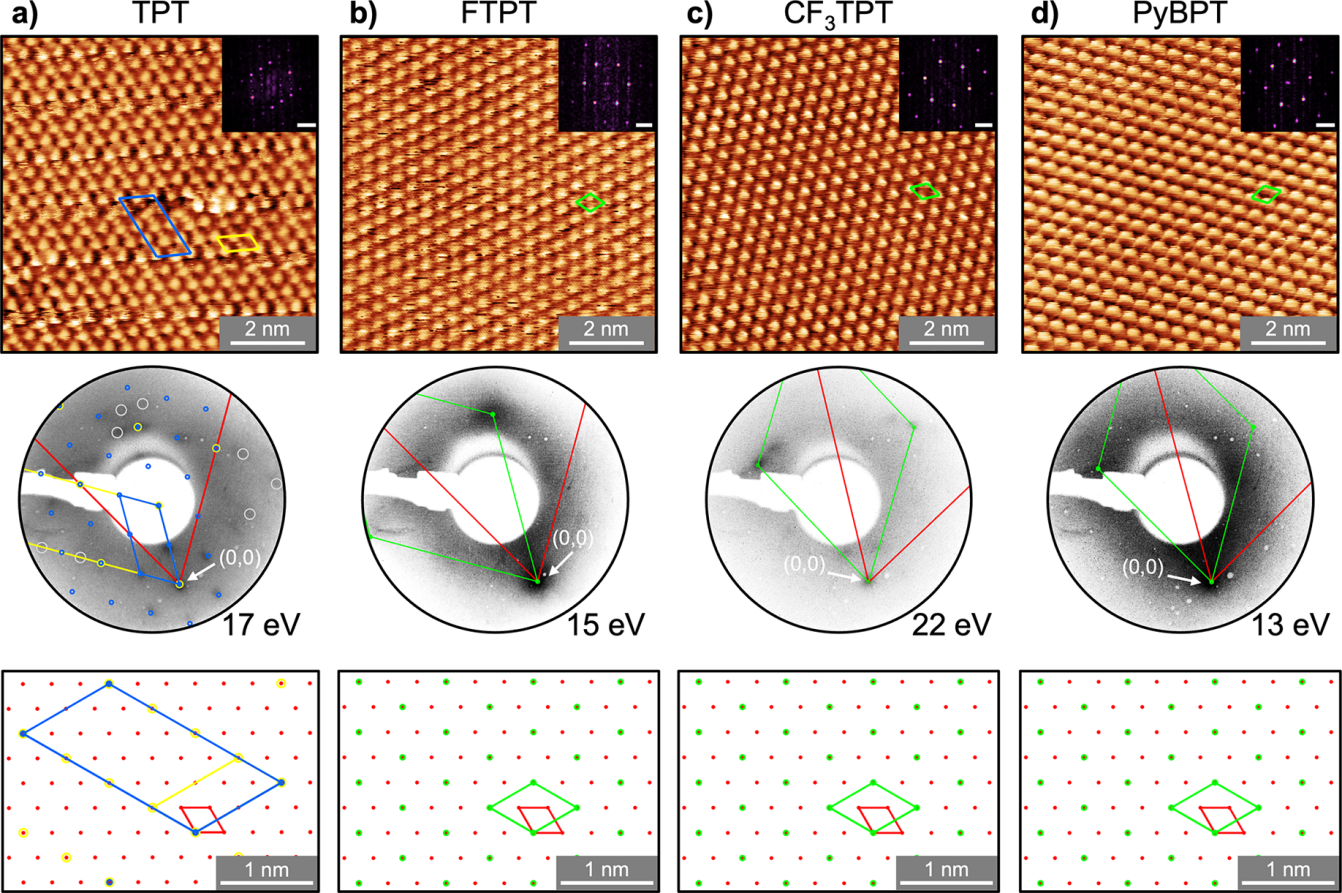
STM data, LEED patterns and the corresponding real space representations of the SAM lattice with respect to the Au(111) surface of a) TPT, b) FTPT, c) CF_3_TPT and d) PyBPT SAMs on Au(111)/mica. All shown STM images are drift-corrected (see Methods). STM conditions: a) −0.7 V, 0.4 nA, 293 K, b)–d) −0.1 V, 1 nA, 293 K. Fast Fourier transform (FFT) of all STM images are shown in the inset (scale bar ≈1 Å^–1^). The unit cells of the molecules (blue, white dashed, green) and Au substrate (red) are highlighted. For the TPT SAM (a) different superstructures were observed in LEED, which are discussed in the text. The yellow highlighted diffraction spot marks the diffraction spot of a $2\sqrt{3}\times \sqrt{3}R30^{{}^{\circ}}$ superstructure, which overlaps with the bigger $2\sqrt{3}\times 4\sqrt{3}R30^{{}^{\circ}}$ ­(blue) superstructure. Diffraction spots, which cannot be described with both $2\sqrt{3}\times \sqrt{3}R30^{{}^{\circ}}$ and $2\sqrt{3}\times 4\sqrt{3}R30^{{}^{\circ}}$ superstructures are highlighted with gray circles. Note that for the PyBPT SAM (d), the LEED diffraction spots appear rather broad, likely due to a low crystalline quality of the Au/mica substrate. The corresponding spatial orientation of the substrate lattice (red) and molecule lattice (blue, yellow, green) in real space are shown in the bottom row.

To further identify the unit cell of the TPT SAM, we studied the samples by LEED (Figure 3a). The LEED pattern obtained at an electron energy of 17 eV shows a 6-fold symmetric structure around the (0,0) reflex. This structure is described with |*a⃗*_1_| = 9.98 ± 0.50 Å, |*a⃗*_2_| = 19.95 ± 0.50 Å, ∠(*a⃗*_1_,*a⃗*_2_) = 120° and forms the corresponding commensurate $2\sqrt{3}\times \sqrt{3}R30^{{}^{\circ}}$ superstructure with respect to the Au(111) surface, confirming the blue unit cell identified by STM. Note that the diffraction spots resulting from the $2\sqrt{3}\times 4\sqrt{3}R30^{{}^{\circ}}$ superstructure overlap with the higher order diffraction spots of the $2\sqrt{3}\times \sqrt{3}R30^{{}^{\circ}}$ superstructure. This can be seen in Figure 3a, where the first-order spots of the smaller $2\sqrt{3}\times \sqrt{3}R30^{{}^{\circ}}$ superstructure (yellow) overlap with the third-order spots of the larger $2\sqrt{3}\times 4\sqrt{3}R30^{{}^{\circ}}$ superstructure (blue). Thus, the LEED pattern agrees with the STM results, however, it does allow to identify the $2\sqrt{3}\times 4\sqrt{3}R30^{{}^{\circ}}$ superstructure unambiguously. Moreover, some of the diffraction spots highlighted with gray could not be assigned, suggesting the presence of an additional arrangement of TPT molecules on Au(111), which was not observed by STM.

For the FTPT, CF_3_TPT, and PyBPT SAMs on Au(111) similar structural results were obtained by both STM and LEED (see Figure 3b–d). The molecular resolved STM data demonstrate highly ordered hexagonal ordering of the molecules for all three SAMs with an intermolecular distance of 5.0 ± 1.0 Å (see Figures S37–S39). The identified unit cells exhibit the lattice parameters |*a⃗*_1_| = |*a⃗*_2_| = 5.0 ± 1.0 Å, ∠(*a⃗*_1_,*a⃗*_2_) = 120° and consist of one molecule showing a $2\sqrt{3}\times \sqrt{3}$ R30° superstructure with respect to the Au(111) surface. The STM based structural analysis, including the FFT results, correlates well with the respective LEED data. Note that, in case of CF_3_TPT SAM (Figure 3c), some minor additional LEED diffraction spots were observed, which are most probably due to coexistence of the $2\sqrt{3}\times 4\sqrt{3}$ R30° superstructure in the sample (see Figure S38b).

Figure 4 presents STM and LEED results for NTPT SAM on Au(111). In contrast to the TPT, FTPT, CF_3_TPT and PyBPT SAMs, two different monolayer structures were observed for this SAM. Thus, an STM image in Figure 4a suggests a densely packed $\sqrt{3}\times \sqrt{3}$ R30° hexagonal molecular arrangement (see also Figure S40a); whereas Figure 4b presents a squared arrangement of the NTPT molecules exhibiting a unit cell (light blue) with lattice parameters |*a⃗*_1_| = (6.9 ± 1.4) Å, |*a⃗*_2_| = (7.3 ± 1.5) Å, ∠(*a⃗*_1_,*a⃗*_2_) = 90° (see also Figure S40b). The FFTs of both superstructures determined from the STM images confirm their hexagonal behavior for the $\sqrt{3}\times \sqrt{3}$ R30° superstructure and the squared structure for the light blue superstructure.

**Figure 4:**
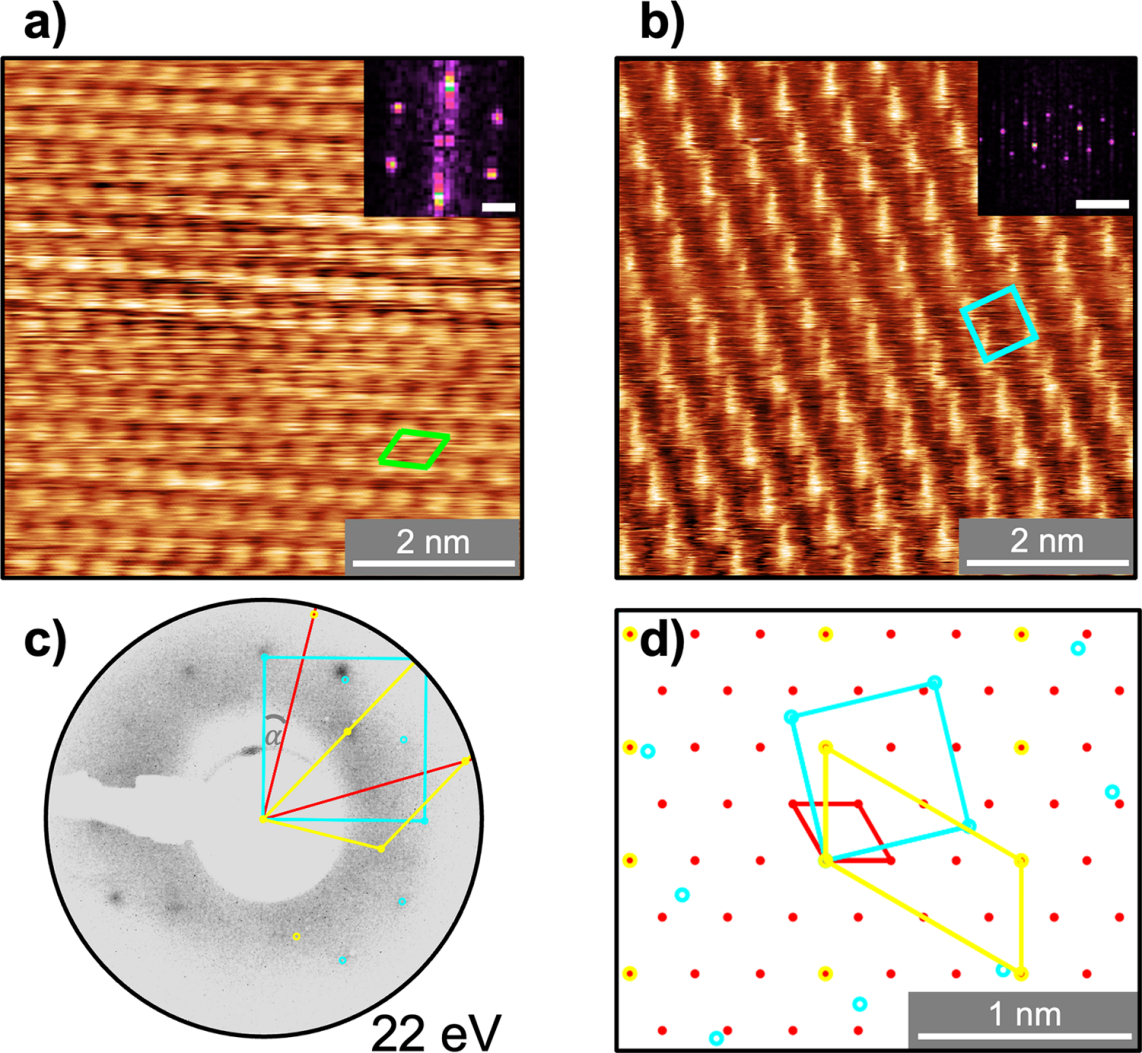
STM data, LEED pattern and the corresponding real space representation of the SAM lattice with respect to the Au(111) surface of NTPT SAM on Au/mica. The shown STM images are drift-corrected (see the Methods). STM conditions: a), b) −0.1 V, 1 nA, 293 K. A fast Fourier transform (FFT) of the STM images a) and b) are shown in the inset (scale bar ≈2 Å^–1^). Highly ordered SAMs were observed both in STM at the molecular level and c) LEED. The unit cells of the NTPT SAM (green, light blue, yellow) and the Au substrate (red) are highlighted. The angle α between the first NTPT lattice vector *a⃗*_1_ and the first substrate lattice vector is highlighted. d) The spatial orientation of the substrate lattice (red) and molecule lattices (light blue, yellow) are shown for both superstructures obtained from LEED in real space in the bottom row.

Next, we analyze both structures observed by STM using the LEED data (Figure 4c and Figure S40c). In Figure 4c the hexagonal unit cell of the Au(111) substrate is highlighted in red. Notably, instead of the from STM expected $\sqrt{3}\times \sqrt{3}$ R30° superstructure a larger $2\sqrt{3}\times \sqrt{3}$ R30° superstructure (yellow) with the lattice parameters of |*a⃗*_1_| = (10.0 ± 1.3) Å, |*a⃗*_2_| = (5.0 ± 1.3) Å, ∠(*a⃗*_1_,*a⃗*_2_) = 120° Two molecules per unit cell can be identified. We attribute this difference to the difficulties with precise identification of the unit cell along with the STM data. Further, the squared molecular arrangement (Figure 4b) observed in STM is consistent with additional diffraction spots in Figure 4c and the unit cell highlighted with light blue. The respective lattice parameters obtained from LEED are |*a⃗*_1_| = |*a⃗*_2_| = 6.5 ± 0.2 Å, ∠(*a⃗*_1_,*a⃗*_2_) = 90°, which correspond well to the STM results and suggest the formation of an incommensurate superstructure with respect to Au(111) most probably with one molecule per unit cell. Moreover, a detailed analysis of all 12 diffraction spots related to this superstructure suggests the presence of three rotational domains. The angle α (gray, Figure 4c) between the first NTPT lattice vector *a⃗*_1_ and the first substrate lattice vector is 15 ± 0.5°. The observed polymorphism of the NTPT SAM is most likely due to the presence of the terminal – NO_2_ group, as STM measurements also revealed the existence of different structural phases for 4′-nitro-1,1′-biphenyl-4-thiol (NBPT) SAMs, in which the aromatic linker is one phenyl ring shorter.^52^

Table 1 sums up the structural features of TPT, FTPT, CF_3_TPT, PyBPT and NTPT SAMs on Au(111) obtained in our study by XPS, STM, and LEED including also available literature data on the molecular tilt angle derived from the near-edge X-ray absorption fine structure (NEXAFS) data. As can be seen, the thicknesses of TPT, FTPT, CF_3_TPT, and PyBPT SAMs obtained from XPS correlate well within the experimental errors with the densely packed arrangements of these molecules on Au(111), corresponding to the area of 21.55 Å^2^ per molecule, and their tilt angles. Considering the observed polymorphism of the NTPT SAMs with two possible surface arrangements corresponding to the molecular areas of either 21.55 Å^2^ or 42.25 Å^2^, the obtained thickness and tilt angle to be considered as some averaged values. Most likely the reported higher tilt angle for the molecules in NTPT SAMs^37^ in comparison to that of TPT,^38^ FTPT,^38^ and CF_3_TPT^38^ SAMs is due to the polymorphic nature of the samples, where the structural phases with high and low molecular density are present simultaneously.

**Table 1:** Summarized Data for TPT, FTPT, CF_3_TPT, PyBPT and NTPT SAMs on Au(111)^a^

SAM	TPT	FTPT	CF_3_TPT	PyBPT	NTPT
*d*_XPS_/Å	13 ± 2	11 ± 2	13 ± 2	13 ± 2	13 ± 2
Unit cell parameters (STM/LEED)	$2\sqrt{3}\times \sqrt{3}$ R30°	$\sqrt{3}\times \sqrt{3}$ R30°	$\sqrt{3}\times \sqrt{3}$ R30°	$\sqrt{3}\times \sqrt{3}$ R30°	$2\sqrt{3}\times \sqrt{3}$ R30°
	$2\sqrt{3}\times 4\sqrt{3}$ R30°				squared
Molecules/unit cell (STM)	2	1	1	1	2
	8				1
Area per molecule/Å^2^	21.55	21.55	21.55	21.55	42.25
	21.55				21.55
Tilt angle backbone (NEXAFS)	19 ± 3 ^38^	21 ± 3 ^38^	13 ± 3 ^38^		25 ± 3 ^37^

a The thicknesses determined by XPS (*d*_XPS_), the unit-cell parameters determined by STM and LEED, and the number of molecules per unit cell are listed. Additionally, the area per molecule is provided. Finally, NEXAFS C K-edge data taken from the literature^37,38^ are shown.

We presented a chemical and structural characterization of the self-assembly down to the nanoscale of five linear three-ring aromatic thiols (Figure 1) on gold. All molecular compounds form chemically well-defined and densely packed SAMs on Au(111) surfaces. The packing density of TPT, FTPT, CF_3_TPT, and PyBPT SAMs is similar and corresponds to the highest possible one on Au(111) with a surface area per molecule of 21.55 Å^2^. Despite the same packing density, there are some structural differences between the monolayers. For the FTPT, CF_3_TPT, and PyBPT SAMs the molecules form a commensurate $\sqrt{3}\times \sqrt{3}$ R30° superstructure with respect to the Au lattice with one molecule per unit cell. However, the molecules in the TPT SAM most probably form two simultaneously existing commensurate superstructures— $2\sqrt{3}\times \sqrt{3}R30^{{}^{\circ}}$ and $2\sqrt{3}\times 4\sqrt{3}R30^{{}^{\circ}}$ —containing two and eight molecules per unit cell, respectively. The NTPT SAMs reveal a simultaneous presence of two structural phases with a higher and lower packing density. One densely packed phase with a commensurate superstructure of $2\sqrt{3}\times \sqrt{3}$ R30° and two molecules per unit cell and a low-density phase forming an incommensurate squared superstructure with one molecule per unit cell and an area per molecule of 42.25 Å^2^. The obtained results provide a solid foundation for applications of the studied molecular systems in nanotechnology for surface modification of gold substrates. Besides that, the possibility to tune the chemical composition and structures of the SAMs make them promising candidates for synthesis of molecular 2D materials with tailored properties *via* the electron irradiation induced cross-linking as was already demonstrated for the TPT SAM.^12^

## 1.1 Materials

1,1′,4′,1″-Terphenyl-4-thiol (TPT, Sigma-Aldrich, 97%) was utilized for SAM preparation. All commercially available chemicals were purchased from Sigma-Aldrich, TCI Germany, VWR International, Fischer Scientific, Carl Roth GmbH & Co., Acros Organics, ABCR, BLDPharm and Alfa Aesar. Anhydrous solvents were dried prior to use on an MBraun SPS-800 system. *N*,*N*-Dimethylformamide (DMF) and ethanol (VWR, HPLC-grade, ≤0.02% H_2_O or <0.1% H_2_O) were used for preparing the SAMs and for rinsing the samples. All chemicals were utilized without further purification. Au/mica substrates (300 nm) with the favored (111) orientation were purchased from Georg Albert PVD.

### 1.1.1 SAM Preparation

For SAM preparation, the utilized glassware was cleaned with peroxymonosulfuric acid (20 min) and rinsed afterward with ultrapure water. The syntheses were performed in an inert atmosphere conducting the Schlenk technique. Au/mica substrates were cleaned directly before utilization with oxygen plasma for 20 s (Diener Zepto). FTPT, CF_3_TPT, PyBPT and NTPT SAMs were synthesized analogous to TPT SAM on Au/mica as reported in the literature.^12^ Here, the precursor molecule was dissolved in DMF. Au/mica substrate was immersed in the reaction mixture. The whole mixture was degassed for 15 min and the synthesis was performed in the dark at 70 °C for 24 h. Afterward all SAMs were rinsed with DMF, EtOH and dried under N_2_ stream.

## 1.2 Methods

*1.2.1. Nuclear Magnetic Resonance Spectroscopy.* NMR data concerning product characterization were collected on BrukerAvance 400 or 600 NEO spectrometers. Chemical shifts (δ) are reported in ppm using residual solvent protons (^1^H NMR: δH = 7.26 ppm for CDCl_3_ and δH = 2.5 ppm for DMSO-*d*_6_, ^13^C NMR: δC = 77.16 for CDCl_3_ and δC = 39.52 ppm for DMSO-*d*_6_) as internal standard. The splitting patterns are designated as follows: s (singlet), d (doublet), dd (doublet of doublets), ddd (doublet of doublets of doublets), t (triplet), dt (doublet of triplets), ddt (doublet of doublets of triplets) and m (multiplet). Coupling constants *J* relate to proton–proton couplings.

*1.2.2. Mass Spectrometry.* High-resolution mass spectrometry (HRMS) was performed using ionization techniques selected according to the solubility and ionization behavior of the respective compounds (see the Supporting Information). Compounds (**5**) and (**7**) were analyzed by high-resolution MALDI-FT-ICR MS on a solariX instrument (Bruker Daltonik GmbH, Bremen, Germany) equipped with a 7.0 T superconducting magnet and an Apollo II Dual ESI/MALDI ion source. For MALDI operation, DCTB (trans-2-[3-(4-*tert*-butylphenyl)-2-methyl-2-propenylidene]­malononitrile) was used as the matrix.

CF_3_TPT (**6**), FTPT (**8**), NTPT (**12**), and compound (**4**) were analyzed by HR-APCI. Samples were prepared by dissolving a small amount of sample (about 0.2 mg) in 1 mL of acetonitrile. The sample solutions were manually injected into an LTQ Orbitrap mass spectrometer (Thermo Fisher Scientific GmbH Bremen, Germany) and measured in positive and negative mode. The sheath gas flow rate was 50, the auxiliary gas flow rate 5, the sweep gas flow rate 5, the spray voltage was set to 5.0 kV. The capillary temperature was 275 °C and the vaporizer temperature was 450 °C.

PyBPT (**3**) was analyzed by HR-ESI-MS using an Agilent 1260 Infinity II 6546 QTOF mass spectrometer, with acetonitrile as solvent.

*1.2.3. X-ray Photoelectron Spectroscopy.* XPS measurements were conducted employing a Scienta Omicron UHV Multiprobe System with a base pressure of <2·10^–10^ mbar. A monochromatic Al K_α_ X-ray source in combination with an electron energy analyzer, Argus CU, with a spectral resolution of 0.6 eV and a photoelectron emission angle with respect to the surface normal θ of 19° was used. Calibration of the XP spectra was performed by utilizing the Au 4f_7/2_ signal at a binding energy of 84.0 eV. The fits were generated with the software CasaXPS^53^ by combining Voigt functions with ratio of Gaussian–Lorentzian functions (30:70, 80:20 for Au 4f) and linear (N 1s, S 2p) or Shirley (O 1s, C 1s, Au 4f) background subtractions. The layer thickness calculations were performed with Beer–Lambert law for the attenuation of the Au 4f_7/2_ signal of the substrate, compared to the signal of a clean gold reference prepared *in situ* by Ar^+^ sputtering.^54^ An inelastic mean free path λ_IMFP_ = 36 Å was used.^55^ The elemental ratios were calculated with the relative sensitivity factors (RSFs) according to Scofield: 1.00, 1.80, 4.43, 1.68, 9.58 for C 1s, N 1s, O 1s, S 2p and Au 4f_7/2_, respectively.^56^ We employed a model assuming a layered structure of the SAM, with the carbon atoms positioned above the thiol bond to the gold substrate. The Beer–Lambert law was applied to account for the attenuation of electrons ejected from the S 2p orbitals.

*1.2.4. Low-Energy Electron Diffraction.* Low-energy electron diffraction (LEED) patterns of the SAMs were obtained using a single microchannel plate (SMCP, Scienta Omicron) LEED system under UHV conditions at room temperature (RT). All images were corrected for geometric distortions and energy errors by LEEDCal software.^57,58^ Subsequently, the corrected images were analyzed with LEEDLab software for quantifying the reciprocal structures of the visible LEED spots.^59^ If at least 7 spots were visible in the recorded LEED pattern, the fit was refined applying a fit algorithm. For the Au(111) surface, lattice parameters of |*a⃗*_1_| = |*a⃗*_2_| = 2.88 Å and ∠(*a⃗*_1_,*a⃗*_2_) = 120° were used.^60^

*1.2.5. Scanning Tunneling Microscopy.* STM measurements were performed in a UHV chamber with pressures of <2·10^–10^ mbar. A VT SPM (variable temperature scanning probe microscopy) system from Scienta Omicron at RT equipped with a W tip was used. The obtained images were evaluated with Gwyddion (Version 2.64).^61^ Drift correction was performed by applying the autocorrelation function and calibrated with the lattice parameters obtained from LEED analysis.

## Supplementary Material



## Notes

Detailed description of the synthesis of 4′-(pyridin-4-yl)-[1,1′-biphenyl]-4-thiol (PyBPT), 4″-(trifluoromethyl)-[1,1′:4′,1″-terphenyl]-4-thiol (CF_3_TPT), 4″-fluoro-[1,1′:4′,1″-terphenyl]-4-thiol (FTPT), 4″-nitro-[1,1′:4′,1″-terphenyl]-4-thiol (NTPT), and other compounds; NMR spectra, MS spectra; XPS data; STM and LEED data (PDF)
