# Supplementary material for: Self-Assembly of Linear Three-Ring Aromatic Thiols on Au(111)
Source: J Phys Chem Lett. 2026 Feb 2;17(6):1838–46. doi: 10.1021/acs.jpclett.5c03949 (PMC12908159; doi:10.1021/acs.jpclett.5c03949)
Supplement: Supplementary file 1 [file jz5c03949_si_001.pdf]

Supporting Information

Self-Assembly of Linear Three-Ring Aromatic Thiols on Au(111)

Verena Müller,[†,#] Anna-Laurine Gaus,[‡,#] Daniel Hüger,[†] Julian Picker,[†] Christof Neumann,[†] Max von Delius,[‡] and Andrey Turchanin[†*]

[†]Institute of Physical Chemistry, Friedrich Schiller University Jena, 07743 Jena, Germany;

[‡]Institute of Organic Chemistry, University of Ulm, 89081 Ulm, Germany

[#]V.M. and A.L.G. contribute equally

*Corresponding author, andrey.turchanin@uni-jena.de

1. Synthesis

Synthesis Pathway A:

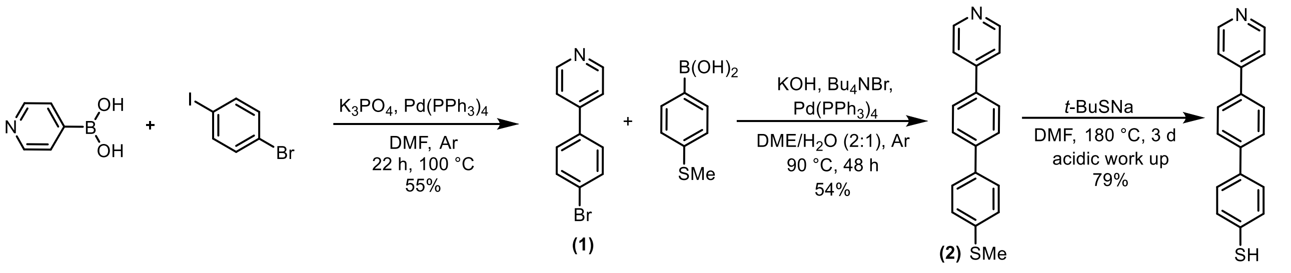

(3) PyBPT

Figure S1. Synthesis of compound **(3) PyBPT.**

Reaction conditions for the molecules synthesized via Pathway A were adapted from previously published literature procedures for biphenylthiols.[1]

Synthesis of 4-(4'-bromophenyl)pyridine (1)

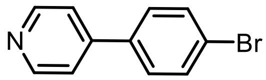

The synthesis was carried out according to the literature.[2] 386 mg (3.14 mmol, 1.00 eq.) of 4-pyridylboronic acid, 1.06 g (3.74 mmol, 1.2 eq.) of 1-bromo-4-iodobenzene and 2.00 g (9.45 mmol, 3.01 eq.) of K_3PO_4 were dissolved in 30 mL of degassed anhydrous DMF. 179 mg (0.15 mmol, 5 mol%) of $Pd(PPh_3)_4$ were added and the reaction mixture was stirred at 100 °C for 22 h in a pressure flask under Ar atmosphere. After cooling to r.t. the crude mixture was extracted with DCM and the combined organic layers were washed with water, before removing the solvent under reduced pressure. The crude product was further purified by column chromatography (dichloro methane/ethyl acetate = 3:1), yielding 405 mg (1.73 mmol, 55%) of the pure product **(1)**. Spectroscopic data are in agreement with the data previously published in the literature.[3]

1H NMR (600 MHz, $CDCl_3$, 298 K): δ = 8.67-8.66 (m, 2H), 7.63-7.61 (m, 2H), 7.51-7.49 (m, 2H), 7.47-7.46 (m, 2H) ppm.

13C NMR (600 MHz, $CDCl_3$, 298 K): δ = 150.6, 147.3, 137.2, 132.5, 128.7, 123.7, 121.5 ppm.

Synthesis of 4-[4'-(methylthio)[1,1'-biphenyl]4-yl]pyridine (2)

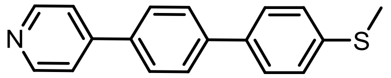

4-(4'-Bromophenyl)pyridine **(1)** (150 mg, 0.64 mmol, 1.0 eq.), 4-(methylthio)phenylboronic acid (107 mg, 0.63 mmol, 1.0 eq.), KOH (0.19 g, 3.3 mmol, 5.2 eq.) and tetrabutylammonium bromide (26 mg, 0.08 mmol, 0.13 eq.) were dissolved in a degassed mixture of 12 mL 1,2-dimethoxyethane and 7 mL water. After degassing the mixture again for 30 min, $Pd(PPh_3)_4$ (27 mg, 0.02 mmol, 3 mol%) was added and the reaction was stirred at 90 °C for 48 h under Ar atmosphere. After cooling to r.t., the solvent was removed under reduced pressure, and the crude residue was dissolved in DCM and washed with water. After evaporating the organic solvent, the crude product was further purified by column chromatography (petroleum spirit/ethyl acetate = 9:1) and yielded 96 mg (0.35 mmol, 54%) of the pure product **(2)**.

1H NMR (600 MHz, $CDCl_3$, 298 K): δ = 8.68 (m, 2H), 7.71 (m, 4H), 7.58-7.57 (m, 4H), 7.35 (m, 2H), 2.54 (s, 3H) ppm.

13C NMR (600 MHz, $CDCl_3$, 298 K): δ = 150.0, 148.5, 141.7, 138.6, 137.0, 136.8, 127.7, 127.6, 127.6, 127.1, 121.7, 15.9 ppm.

HR MS (ESI) positive mode: Calculated for $C_{18}H_{16}NS^+$: 278.0998 m/z. Found: 278.1004 m/z.

Synthesis of 4-(4'-(methylthio)-[1,1'-biphenyl]-4-yl)pyridine PyBPT (3)

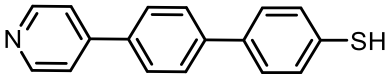

30 mg (0.11 mmol) of compound **(2)** and 31 mg (0.27 mmol, 2.7 eq.) sodium 2-methyl-2-propanethiolate were dissolved in 5 mL anhydrous DMF and heated to 170 °C for 3 days. The solution was cooled down to r.t. and an aqueous H_2SO_4 solution was added until pH 6 was reached and a white precipitate formed. The mixture was extracted with DCM, and the solvent of the combined organic layers were removed under reduced pressure. The crude NMR showed pure compound. Yield: 30 mg (0.11 mmol, 100%). To remove small grease residues, the solid was first dissolved in chloroform and then precipitated by adding n-pentane. After centrifugation, the solution was carefully removed, and the precipitate was again dissolved in chloroform. This procedure was done 3-4 times and decreased the yield to 23 mg (0.087 mmol, 79%).

1H NMR (600 MHz, $CDCl_3$): δ = 8.68 (d, ^3J = 5.2 Hz, 2H), 7.73-7.68 (m, 4H), 7.55 (d, ^3J = 5.2 Hz, 2H), 7.52 (d, ^3J = 8.2 Hz, 2H), 7.37 (d, ^3J = 8.2 Hz, 2H), 3.52 (s, 1H) ppm.

13C NMR (600 MHz, $CDCl_3$): δ = 150.4, 148.0, 141.3, 137.8, 137.1, 130.8, 130.0, 127.8, 127.6, 127.6, 121.6 ppm.

HR MS (ESI) positive mode: Calculated for $C_{17}H_{14}NS^+$: 264.0841 m/z. Found: 264.0823 m/z.

Synthesis Pathway B:

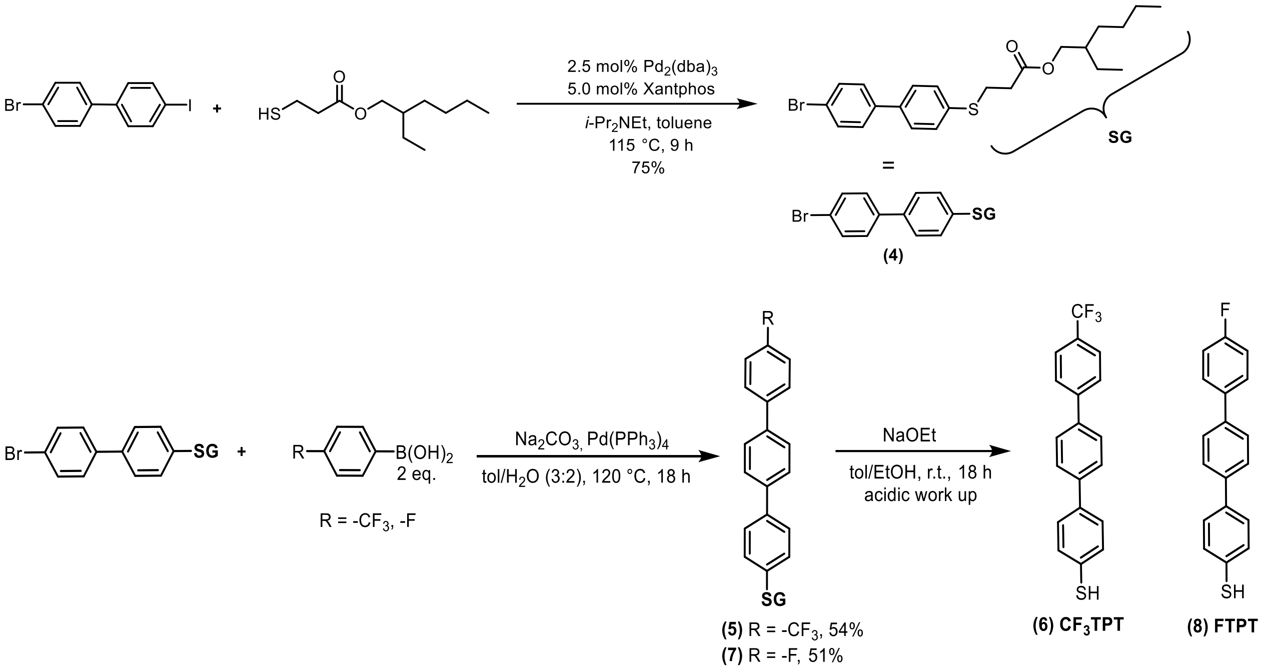

Figure S2. Synthesis of compound **(6) CF_3TPT** and **(8) FTPT.**

Reaction conditions for the molecules synthesized via Pathway B were adapted from previously published literature procedures for biphenylthiols.[4]

3-(4’-Bromobiphenyl-4-sulfanyl)propionic acid 2-Ethylhexyl Ester (4)

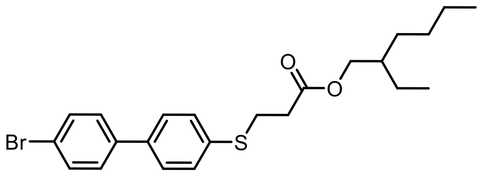

After dissolving 2.00 g (5.59 mmol, 1.0 eq.) 4'-bromo-3-iodo-1,1'-biphenyl in 25 mL anhydrous toluene, *i*-Pr_2NEt (2.0 mL, 11.5 mmol, 2.1 eq.) was added, and the mixture was evacuated and back-filled with argon three times. 162 mg (0.28 mmol, 5.0 mol%) of Xantphos, 130 mg (0.14 mmol, 2.5 mol%) of $Pd_2(dba)_3$ and 2-ethylhexyl-3-mercaptopropionate (1.3 mL, 5.7 mmol, 1.0 eq.) was added, and then the mixture was degassed twice more. The reaction mixture was stirred at 115 °C for 9 h. After removing the solvent under reduced pressure, the product was purified by column chromatography with petroleum spirit/dichloromethane (2:1) yielding 1.90 g (4.23 mmol, 75%) of the product compound **(4)**. Minor impurities marked with *in the respective NMR spectra belonging to an aromatic side product. The product was not further purified and was used directly for the subsequent cross coupling reactions.

1H NMR (600 MHz, $CDCl_3$, 298 K): δ = 7.57-7.54 (m, 2H), 7.49-7.47 (m, 2H), 7.44-4.40 (m, 4H), 4.05-3.99 (m, 2H), 3.21 (t, ^3J = 7.4 Hz, 2H), 2.66 (t, ^3J = 7.4 Hz, 2H), 1.56 (m, 1H), 1.38-1.28 (m, 8H), 0.90-0.89 (t, ^3J = 7.3 Hz, 6H) ppm.

13C NMR (151 MHz, $CDCl_3$, 298 K): δ = 172.0, 139.4, 138.3, 135.2, 132.1, 130.3, 128.6, 127.6, 121.8, 67.4, 38.9, 34.6, 30.5, 29.1, 29.1, 23.9, 23.1, 14.2, 11.1 ppm.

Impurities at: 1H NMR (600 MHz, $CDCl_3$): δ = 7.76, 7.30 ppm.

HRMS (APCI): Calculated for $C_{23}H_{29}BrO_2S$: 448.1072 m/z. Found: 448.1049 m/z.

Synthesis of compound (5)

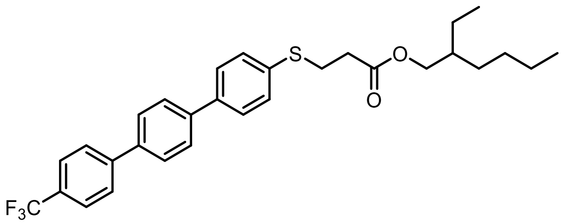

250 mg (0.56 mmol, 1 eq.) of compound **(4)** was dissolved in a degassed mixture of 6 mL toluene and 4 mL of water. 4-(Trifluoromethyl)phenyl boronic acid (211 mg, 1.1 mmol, 2 eq.) and Na_2CO_3 (234 mg, 2.2 mmol, 4 eq.) were added and the mixture was evacuated and backfilled with argon three times. $Pd(PPh_3)_4$ (43 mg, 0.04 mmol, 6.6 mol%) was added and the mixture was stirred at 120 °C over night.

After extraction with ethyl acetate and water, the solvent was removed under reduced pressure. The crude product was purified by coloumn chromatography using a cyclohexane/ethyl acetate (95:5) solvent mixture yielding 156 mg (0.3 mmol) 54% of the pure compound.

1H NMR (600 MHz, $CDCl_3$, 298 K): δ = 7.72 (m, 4H), 7.68 (s, 4H), 7.59-7.57 (m, 2H), 7.58 (m, 2H), 7.46-7.44 (m, 2H), 4.05-4.00 (m, 2H), 3.23 (t, ^3J = 7.4 Hz, 2H), 2.68 (t, ^3J = 7.4 Hz, 2H), 1.60-1.55 (m, 1H), 1.38-1.24 (m, 8H), 0.90-0.88 (m, 6H) ppm.

13C NMR (151 MHz, $CDCl_3$, 298 K): δ = 172.0, 144.3, 140.4, 138.9, 138.7, 135.1, 130.4, 127.9, 127.7, 127.6, 127.4, 125.9 (d), 123.5, 67.4, 38.9, 34.6, 30.6, 29.2, 29.1, 23.9, 23.1, 14.2, 11.2 ppm.

19F NMR (376 MHz, $CDCl_3$, 298 K): δ = -62.4 ppm.

HRMS (MALDI): Calculated for $C_{30}H_{33}F_3O_2S$: 514.2153 m/z. Found: 514.2145 m/z.

Synthesis of CF_3TPT (6)

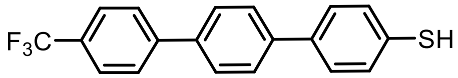

Compound **(7)** (20 mg, 0.04 mmol) of was dissolved in 2.5 mL of anhydrous toluene. 0.2 mL (0.5 mmol, 21 wt% in EtOH) sodium ethoxide and 2 mL of anhydrous EtOH were added and the mixture was stirred for 18 h at r.t. The reaction mixture was acidified with an aqueous citric acid solution until pH 7 was reached and stirred for additional 90 min. After extraction with ethyl acetate, the combined organic layers were washed with water, the volatiles were removed under reduced pressure, yielding 16 mg (0.04 mmol, 100%) of the pure compound.

1H NMR (600 MHz, $CDCl_3$): δ = 7.72 (m, 4H), 7.67 (m, 4H), 7.52 (d, ^3J = 8.2 Hz, 2H), 7.37 (d, ^3J = 8.2 Hz, 2H), 3.52 (s, 1H) ppm.

13C NMR (151 MHz, $CDCl_3$, 298 K): δ = 144.3, 140.4, 138.8, 138.0, 130.5, 130.0, 129.5, 127.9, 127.8, 127.5, 127.4 125.9 (d), 29.9 ppm.

19F NMR (376 MHz, $CDCl_3$, 298 K): δ = -62.4 ppm.

HRMS (APCI): Calculated for $C_{19}H_{12}F_3S$ $[M-H]^-$: 329.0612 m/z. Found: 329.0568 m/z.

Synthesis of compound (7)

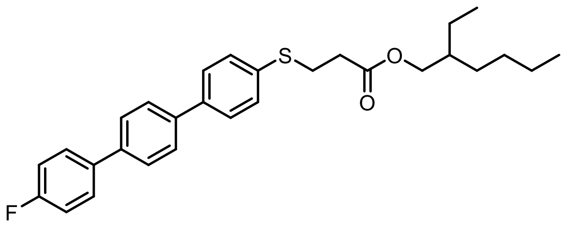

259 mg (0.57 mmol, 1 eq.) of compound **(4)** was dissolved in a degassed mixture of toluene (6 mL) and water (4 mL). Then 4-Fluorophenyl boronic acid (157 mg, 1.12 mmol, 2 eq.) and Na_2CO_3 (242 mg, 2.28 mmol, 4 eq.) were added and the mixture was evacuated and back-filled with nitrogen three times. $Pd(PPh_3)_4$ (50 mg, 0.04 mmol, 7 mol%) was added and the mixture was stirred at 110 °C for 18 h. After cooling to r.t., the crude mixture was extracted with ethyl acetate and the combined organic layers were washed with water. Evaporation of the solvent followed by purification *via* coloumn chromatography (cylohexane/ ethyl acetate = 95:5) yielded 137 mg (0.29 mmol, 51%) of the pure solid compound **(7)**.

1H NMR (400 MHz, $CDCl_3$, 298 K): δ = 7.64-7.56 (m, 8H), 7.44 (m, 2H), 7.15 (m, 2H), 4.02 (m, 2H), 3.23 (t, J = 7.4 Hz, 2H), 2.68 (t, J = 7.4 Hz, 2H), 1.42-1.26 (m, 9H), 0.90 (t, J = 7.4 Hz, 6H) ppm.

13C NMR (151 MHz, $CDCl_3$, 298 K): δ = 172.0, 162.7 (d, $^1J_{C\text{-}F}$ = 246.8 Hz), 139.4, 139.4, 139.0, 136.9 (d, $^4J_{C\text{-}F}$ = 3.2 Hz), 134.8, 130.5, 128.7 (d, $^3J_{C\text{-}F}$ = 8.1 Hz), 127.7, 127.6, 127.4, 115.9 (d, $^2J_{C\text{-}F}$ = 21.4 Hz), 67.4, 38.9, 34.6, 30.6, 29.3, 29.1, 23.9, 23.1, 14.2, 11.1 ppm.

19F NMR (376 MHz, $CDCl_3$, 298 K): δ = -115.5 ppm.

HRMS (MALDI): Calculated for $C_{29}H_{33}FO_2S$: 464.2185 m/z. Found: 464.2182 m/z.

Synthesis of FTPT (8)

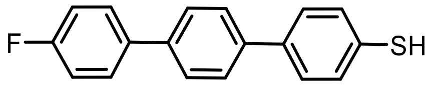

87 mg (0.19 mmol) of compound **(7)** were dissolved in 2 mL of toluene and 4 mL of ethanol. 0.2 mL (0.5 mmol, 21 wt% in EtOH) sodium ethoxide were added under Ar and the reaction was stirred at r.t. for 17 h. The reaction mixture was acidified with an aqueous citric acid solution until pH 6 was reached and stirred for additional 1.5 h. After extraction with ethyl acetate, the combined organic layers were washed with water, the volatiles were removed under reduced pressure and crude product was purified by column chromatography using cyclohexane/ethyl acetate 95:5 yielding 18 mg (0.06 mmol, 31%) of the pure compound **(8)**.

1H NMR (600 MHz, $CDCl_3$, 298 K): δ = 7.64-7.56 (m, 6H), 7.52-7.50 (m, 2H), 7.37-7.35 (m, 2H), 7.16-7.12 (m, 2H), 3.51 (s, 1H) ppm.

13C NMR (151 MHz, $CDCl_3$, 298 K): δ = 162.7 (d, $^{1}J_{C-F}$ = 246.7 Hz), 139.4, 138.2, 136.9 (d, $^{4}J_{C-F}$ = 3.2 Hz), 130.2, 130.0, 128.7 (d, $^{3}J_{C-F}$ = 7.8 Hz), 127.7, 127.6, 127.4, 115.9 (d, $^{2}J_{C-F}$ = 21.4 Hz) ppm.

19F NMR (376 MHz, $CDCl_3$, 298 K): δ = -115.5 ppm.

HRMS (APCI): Calculated for $C_{18}H_{12}FS$: 279,0644 m/z. Found: 279.0580 m/z.

Synthesis Pathway C:

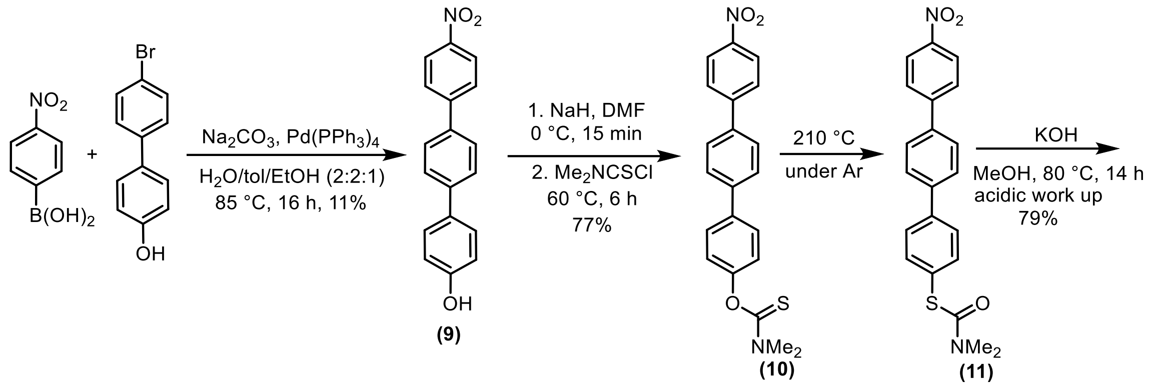

Figure S3. Synthesis Overview for compound **(9)-(12)** as previously described in literature.[5]

Synthesis of 4''-nitro-4-hydroxyterphenyl (9)

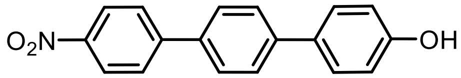

Compound **(9)** was synthesized following the reported procedure[5] with minor modifications regarding the solvent mixture for coloumn chromatography. 4-Nitrophenylboronic acid (2.500 g, 14.9 mmol, 1.1 eq.) was suspended in a mixture of 12 mL EtOH and 24 mL H_2O and degassed for 45 min. 3.273 g (13.1 mmol) 4-Bromo-4'-hydroxybiphenyl, 5.054 g (47.6 mmol, 3.6 eq.) of Na_2CO_3 and 22 mL of degassed toluene was added to the mixture. After the addition of 504 mg (0.4 mmol, 3 mol%) $Pd(PPh_3)_4$ the mixture was heated to 85 °C for 16 h. After cooling to r.t., the reaction was extracted with ethyl acetate and washed with brine. After removing the solvent of the organic phase under reduced pressure, the product was purified by column chromatography starting with a 1:1 solvent mixture of toluene/DCM and increasing the DCM amount stepwise until a 1:4 ratio was reached. The purification yielded 423 mg (1.45 mmol, 11%) of compound **(11)**. The NMR data are in agreement with the previously reported data in literature.[5]

1H NMR (600 MHz, DMSO-*d6*, 298 K): δ = 9.64, 8.32-8.30i (m, 2H), 8.02-8.00(m, 2H), 7.86-7.84 (m, 2H), 7.76-7.74 (m, 2H), 7.60-7.57 (m, 2H), 6.89z-6.86 (m, 2H) ppm.

Synthesis of compound (10)

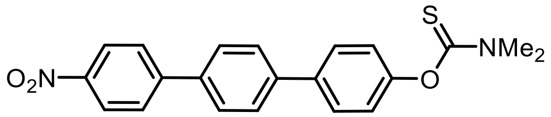

73 mg (1.8 mmol, 1.5 eq., 60% dispersion in paraffin oil) sodium hydride was washed with 1.4 mL dry n-pentane under inert atmosphere at 0 °C. A solution of 348 mg (1.2 mmol, 1 eq.) 4’’-Nitro-4-hydroxyterphenyl **(9)** in 3 mL anhydrous DMF and additional 11 mL of anhydrous DMF were added at 0 °C and the reaction was stirred for 15 min. The mixture was allowed to warm up to r.t., stirred for additional 30 min at r.t., and was then heated to 60 °C for 6 h. After cooling again to r.t., the reaction was quenched by slow addition of water, extracted with DCM and washed with brine. After removing the organic solvent under reduced pressure, the product was recrystallized from a 1:1 mixture of ethanol/ethyl acetate, and yielding 350 mg (0.92 mmol, 77%) of the pure compound **(10)**. The NMR data are in agreement with the previously reported data in literature.[5]

1H NMR (400 MHz, DMSO-d6, 298 K): δ = 8.35-8.31 (m, 2H), 8.06-8.03 (m, 2H), 7.93-7.90 (m, 2H), 7.88-7.85 (m, 2H), 7.80-7.77 (m, 2H), 7.21-7.17 (m, 2H), 3.38 (s, 3H), 3.34 (s, 3H) ppm.

Synthesis of compound (11) *via* Newman-Kwart Rearrangement

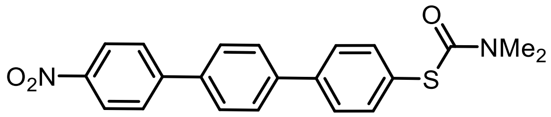

A Kugelrohr apparatus was flushed with argon and a small glass vial containing compound **(10)** (24.7 mg, 0.07 mmol) was carefully placed inside. The apparatus was heated to 210 °C overnight. The resulting brownish product **(11)** was used without further purification (23.9 mg, 0.06 mmol, 97%). The NMR data are in agreement with the previously reported data in literature.[5]

^{1}H NMR (400 MHz, DMSO-d$_6$, 298 K): δ = 8.34-8.31 (m, 2 H), 8.06-8.03 (m, 2 H), 7.94-7.92 (m, 2H), 7.89-7.87 (m, 2 H), 7.81-7.79 (m, 2H), 7.56-7.54 (m, 2H), 3.07 (br s, 3 H), 2.95 (br s, 1H) ppm.

Synthesis of NTPT (12)

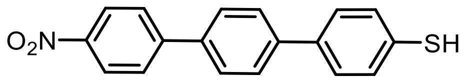

KOH (225 mg, 4 mmol) was dissolved in 2 mL MeOH and degassed for 20 min. The solution was added to 100 mg (0.26 mmol) of compound **(11)** and the suspension was heated to 80 °C for 14 h under argon. An aqueous solution of HCl was degassed and added dropwise to the reaction mixture at r.t. yielding a pale yellow precipitate. After stirring for additional 30 min, the precipitate was filtered, washed with water and the solid was dried under reduced pressure yielding 63 mg (20.5 mmol, 79%) of the yellow product.

1H NMR (600 MHz, $CDCl_3$, 298 K): δ = 8.33-8.31 (m, 2H), 7.79-7.77 (m, 2H), 7.74-7.68 (m, 2H), 7.54-7.51 (m, 2H), 7.39-7.37 (m, 2H), 3.52 (s, 1H) ppm.

13C NMR (151 MHz, $CDCl_3$, 298 K): δ = 147.3, 147.2, 141.1, 137.8, 137.7, 130.9, 130.0, 128.0, 127.8, 127.8, 127.7, 124.4 ppm.

HRMS (APCI): Calculated for $C_{18}H_{12}NO_2S$ $[M-H]^-$: 306.0594 m/z. Found: 306.0529 m/z.

SPECTRA:

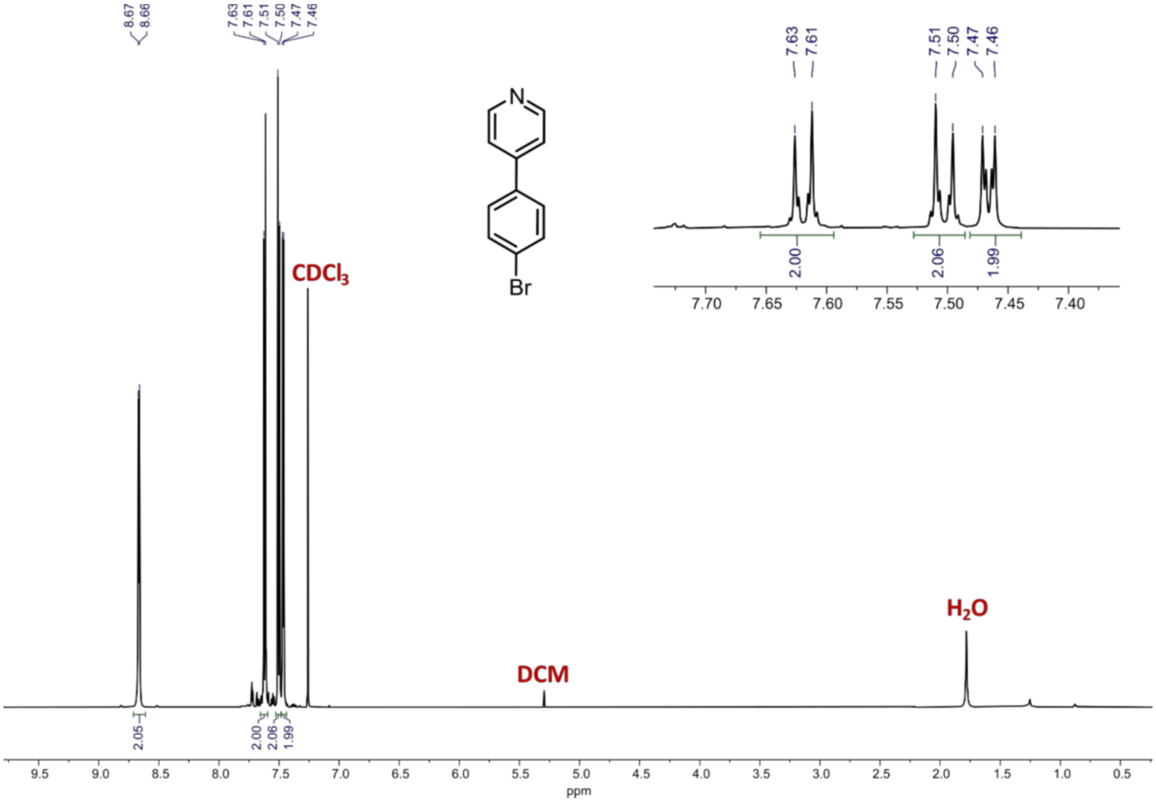

Figure S4. ^{1}H NMR spectrum (600 MHz, 298 K, in $CDCl_3$) of compound **(1)**.

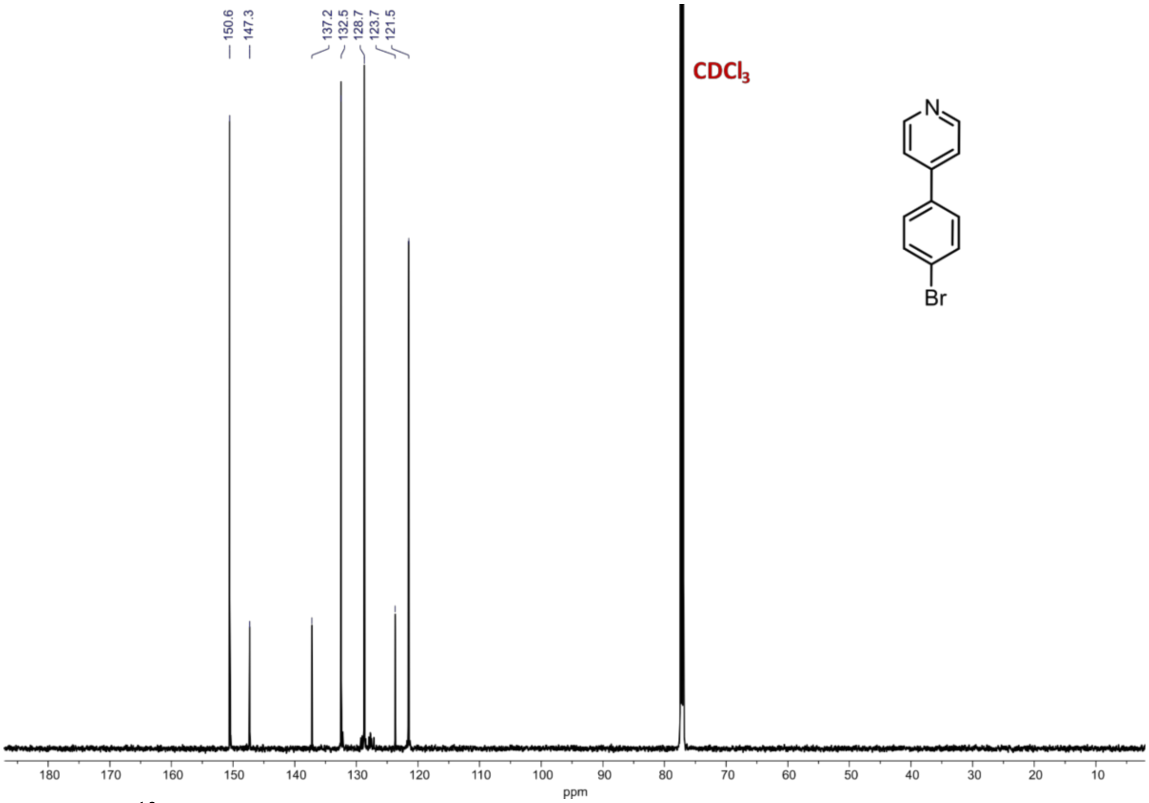

Figure S5. ^{13}C NMR spectrum (151 MHz, 298 K, $CDCl_3$) of compound **(1).**

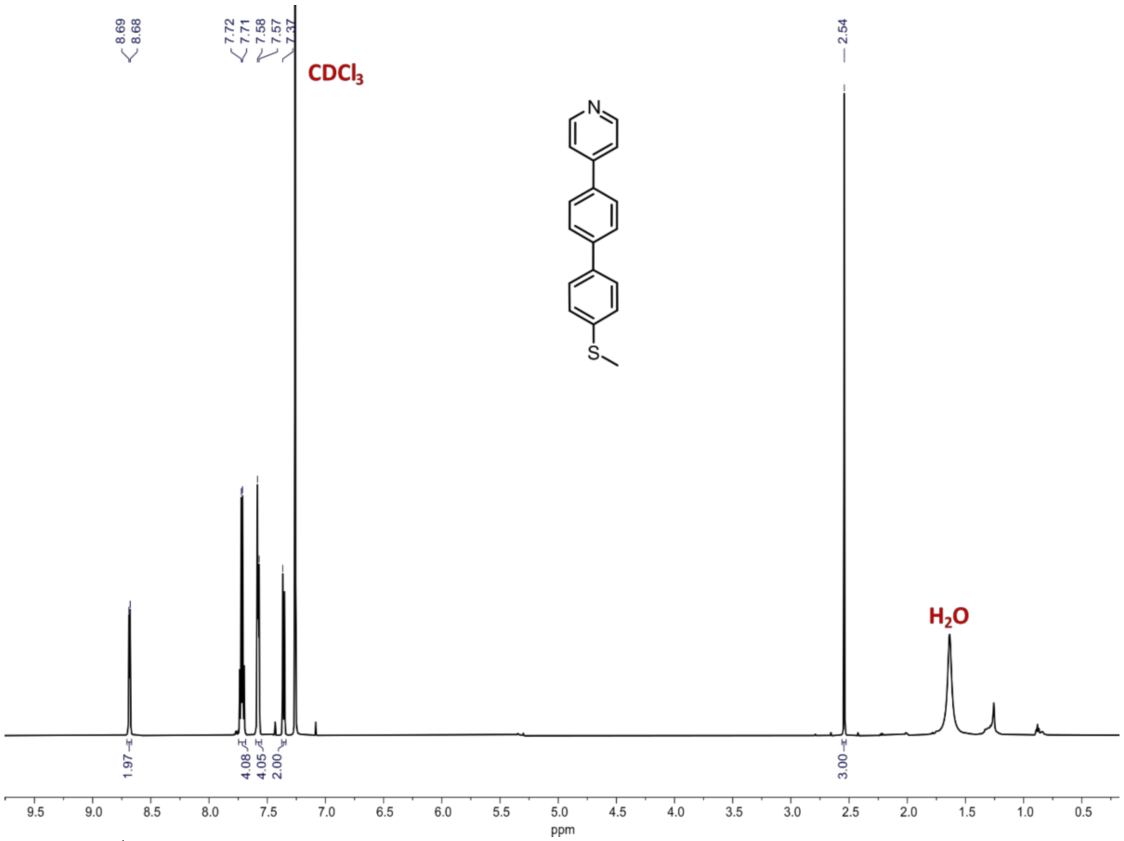

Figure S6. ^{1}H NMR spectrum (600 MHz, 298 K, $CDCl_3$) of compound **(2)**.

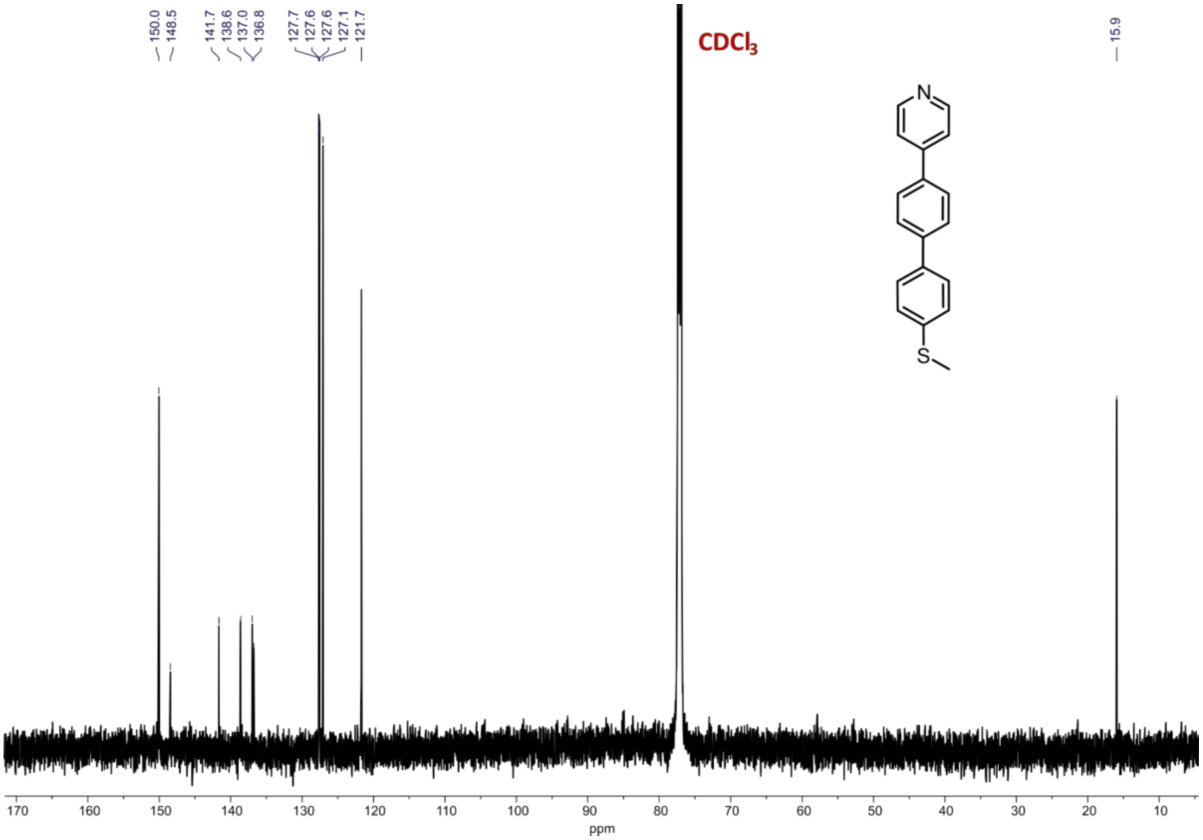

Figure S7. 13C NMR spectrum (151 MHz, 298 K, CDCl3) of compound **(2)**.

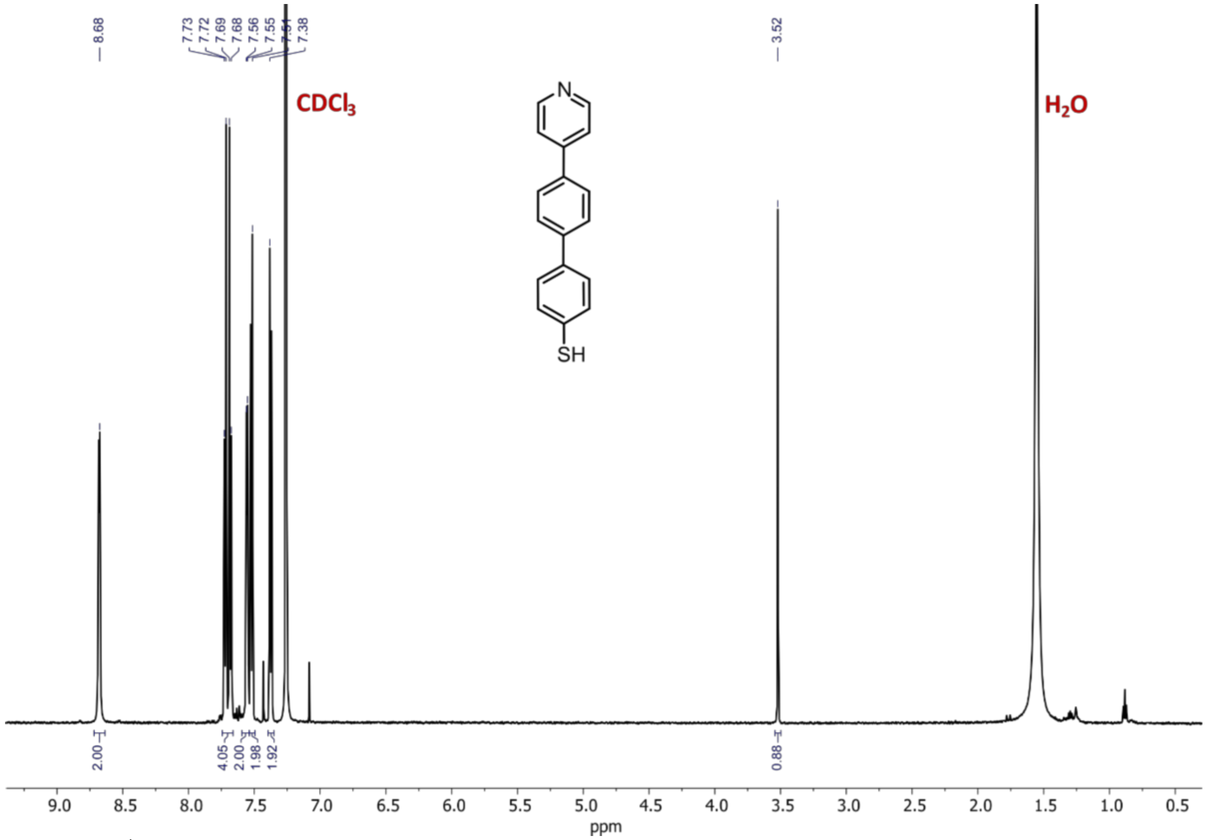

Figure S8. ^{1}H NMR spectrum (600 MHz, 298 K, $CDCl_3$) of compound **(3)**.

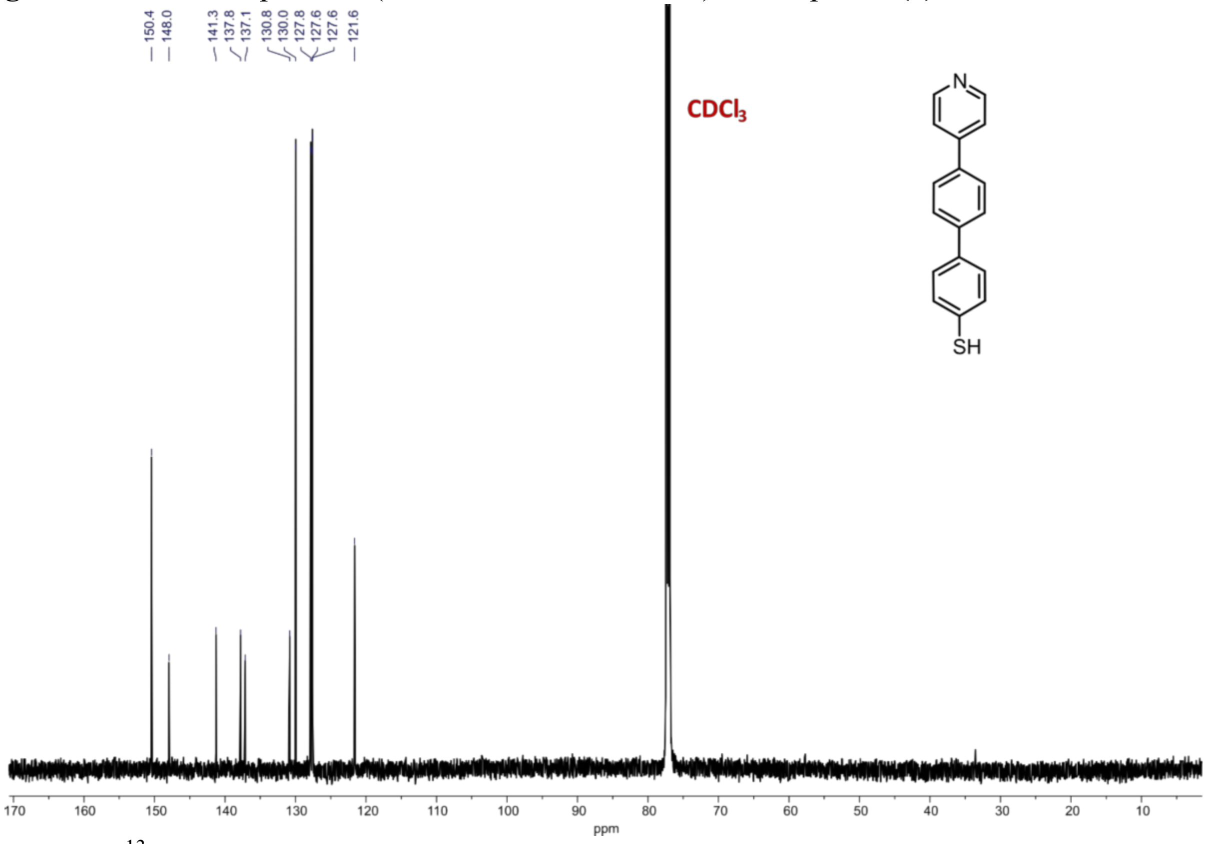

Figure S9. ^{13}C NMR spectrum (151 MHz, 298 K, $CDCl_3$) of compound **(3)**.

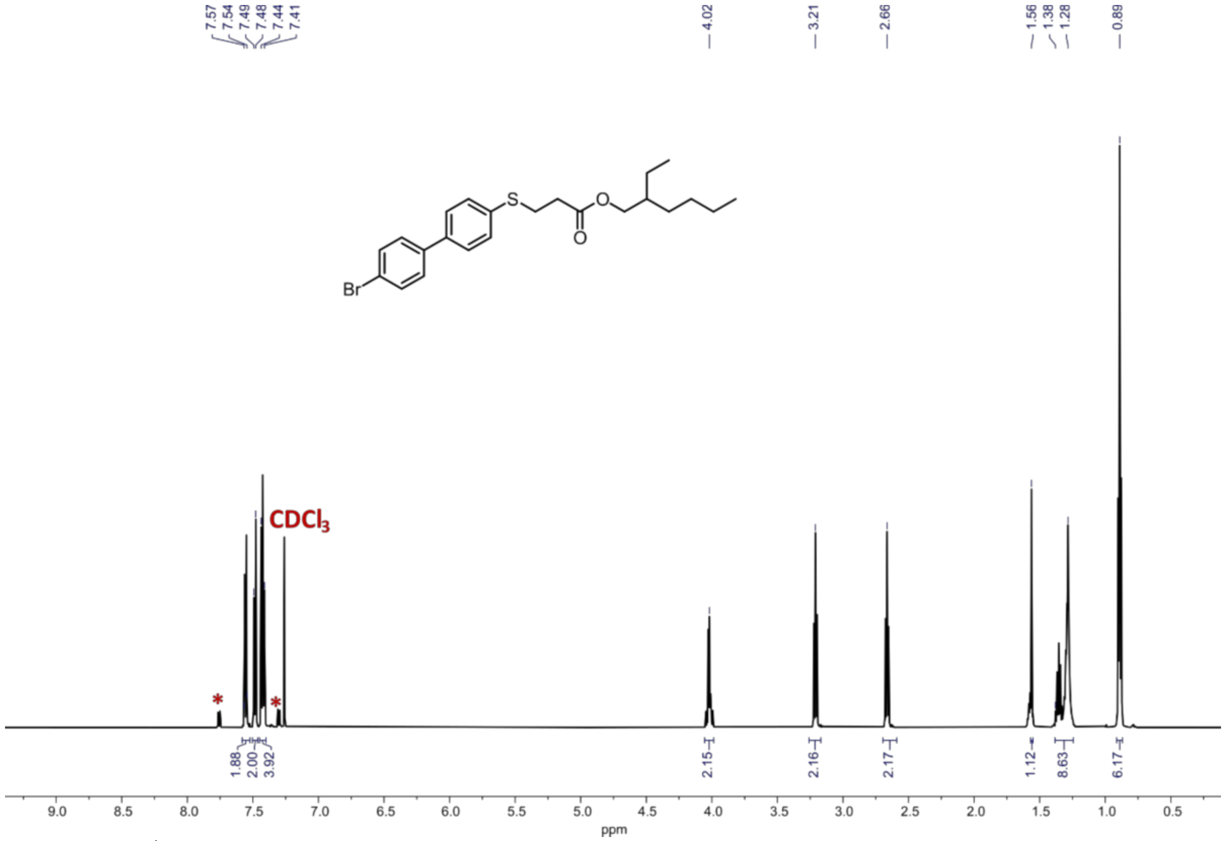

Figure S10. ^{1}H NMR spectrum (600 MHz, 298 K, in $CDCl_3$) of compound **(4).**

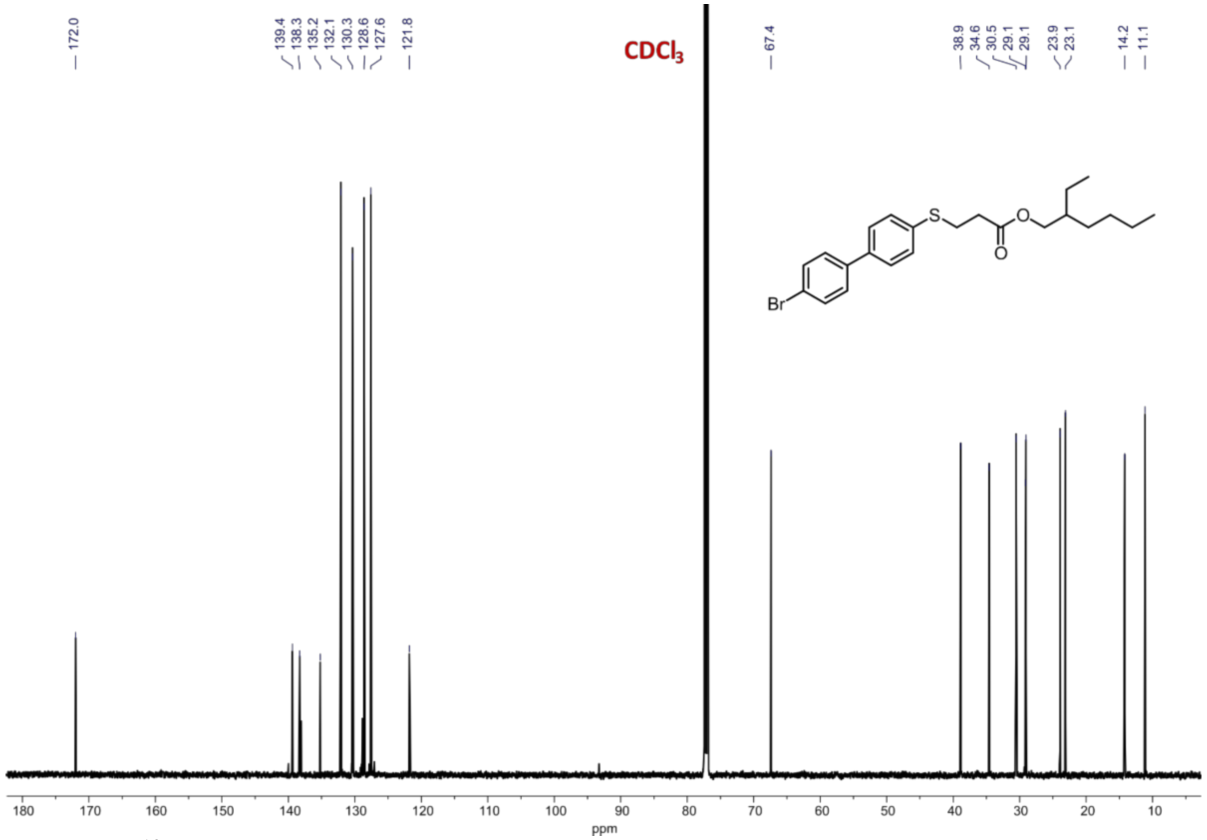

Figure S11. ^{13}C NMR spectrum (151 MHz, 298 K, $CDCl_3$) of compound **(4)**.

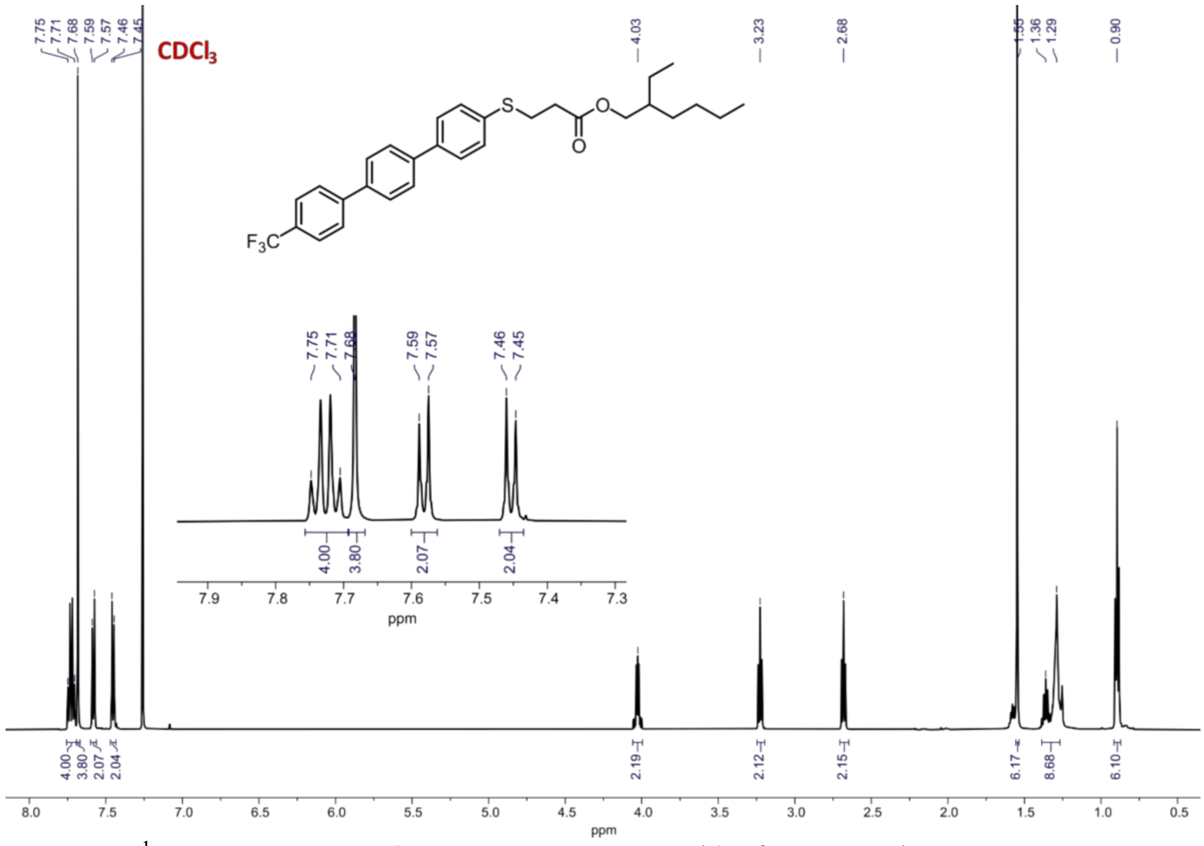

Figure S12. ^{1}H NMR spectrum (600 MHz, 298 K, $CDCl_3$) of compound **(5)**.

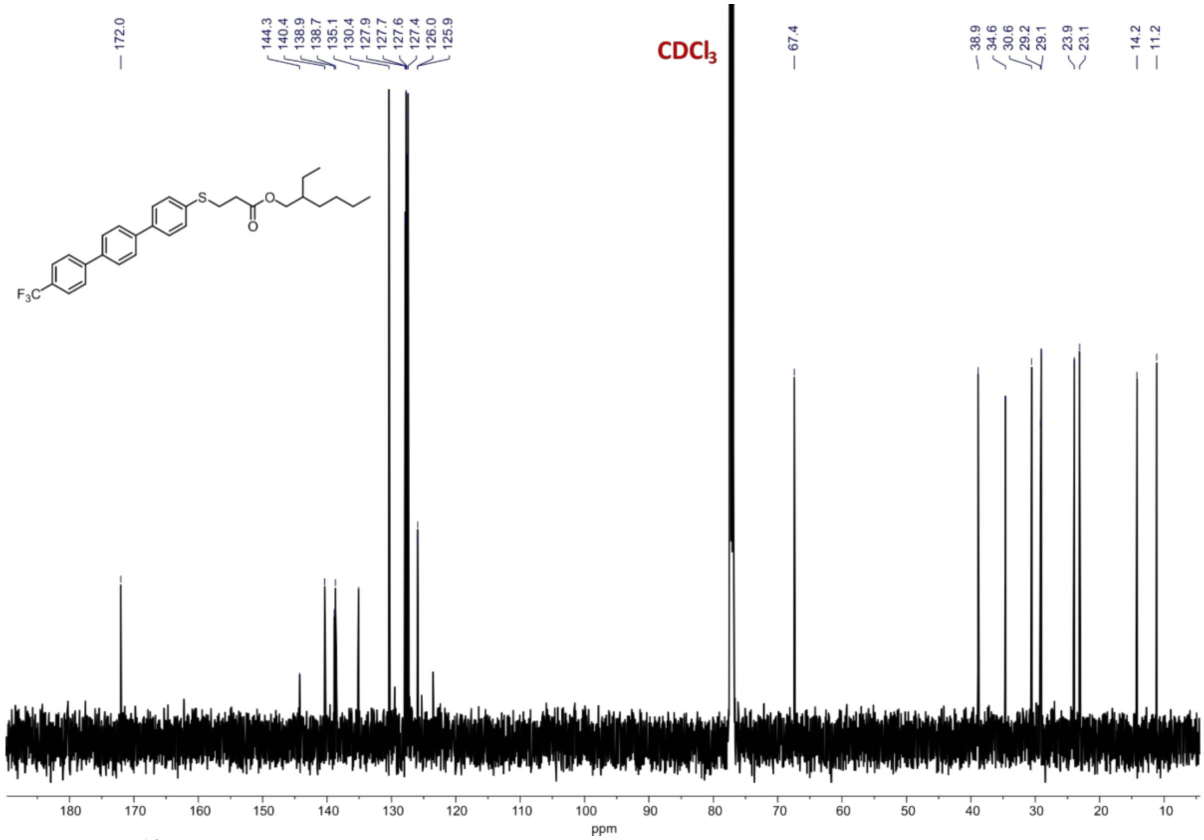

Figure S13. ^{13}C NMR spectrum (151 MHz, 298 K, $CDCl_3$) of compound **(5)**.

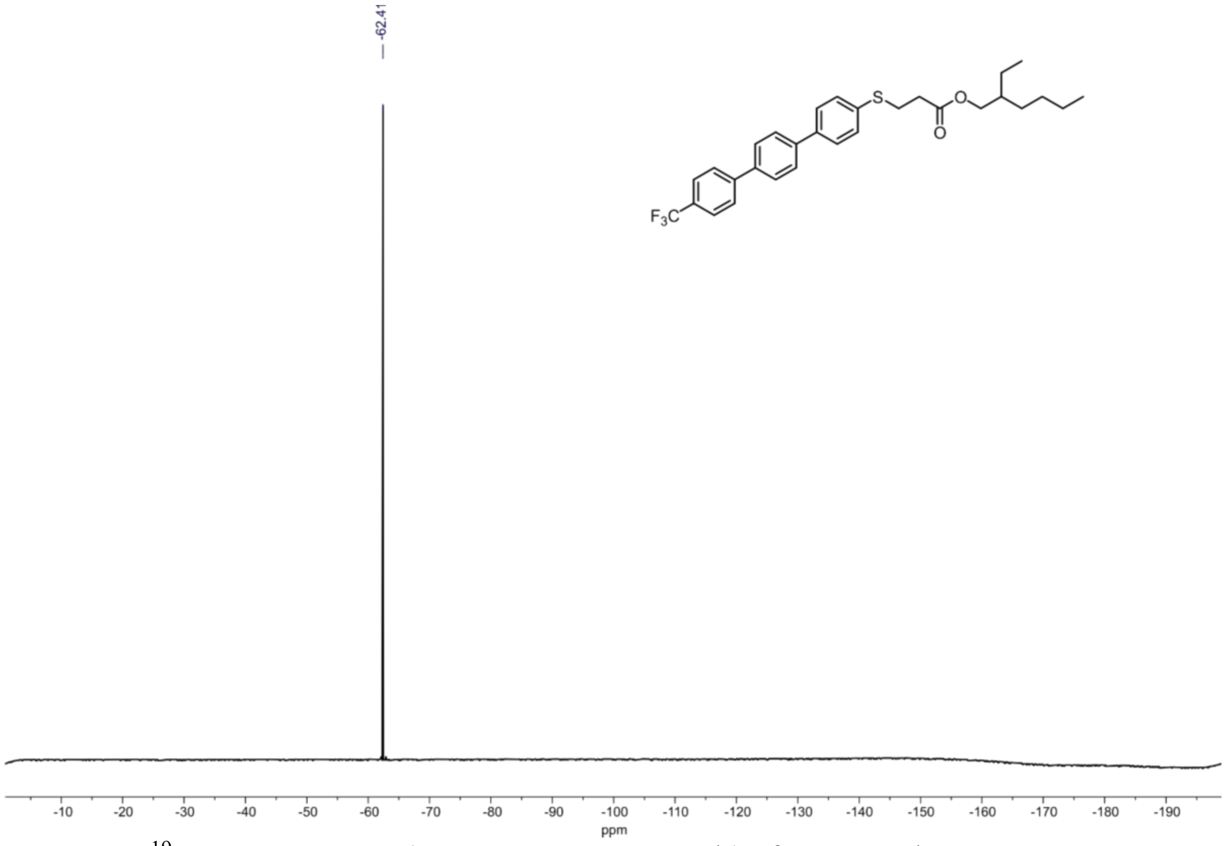

Figure S14. ^{19}F NMR spectrum (376 MHz, 298 K, $CDCl_3$) of compound **(5)**.

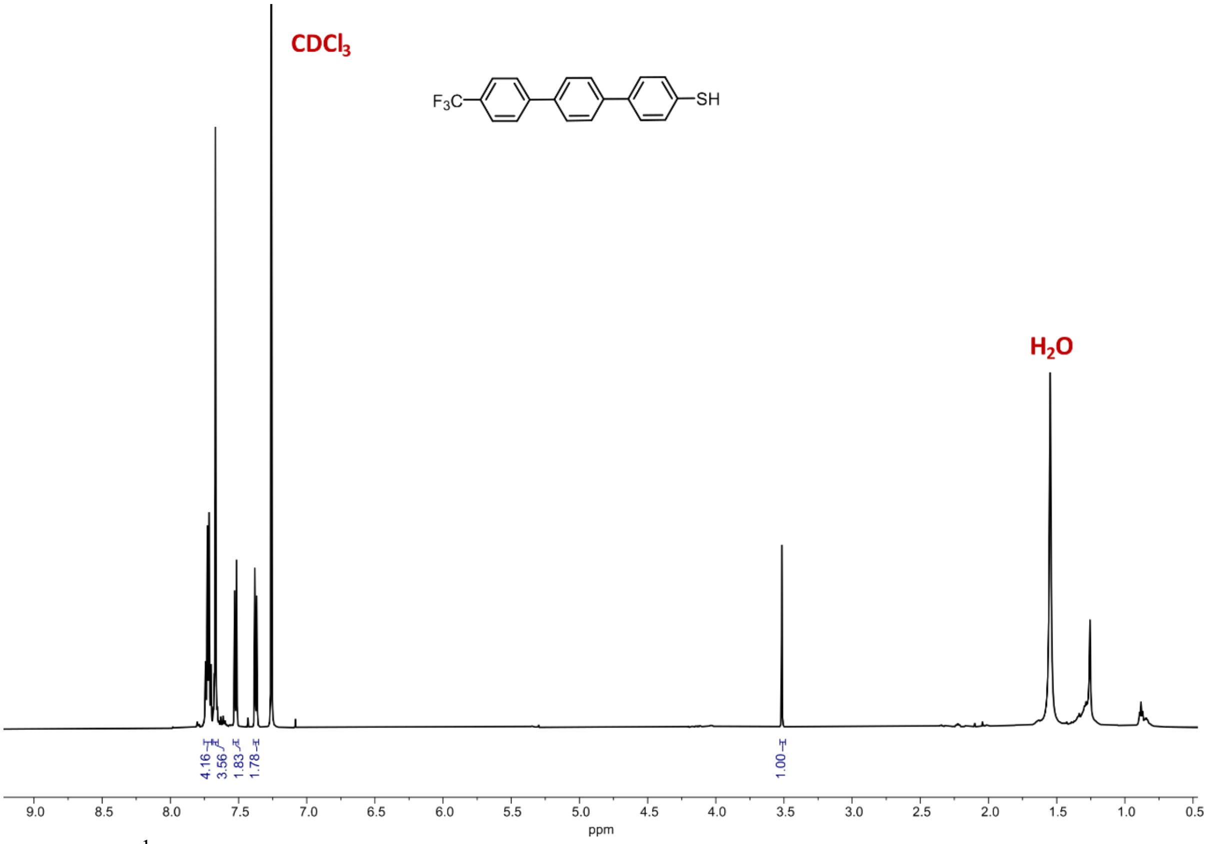

Figure S15. ^{1}H NMR spectrum (600 MHz, 298 K, $CDCl_3$) of compound **(6).**

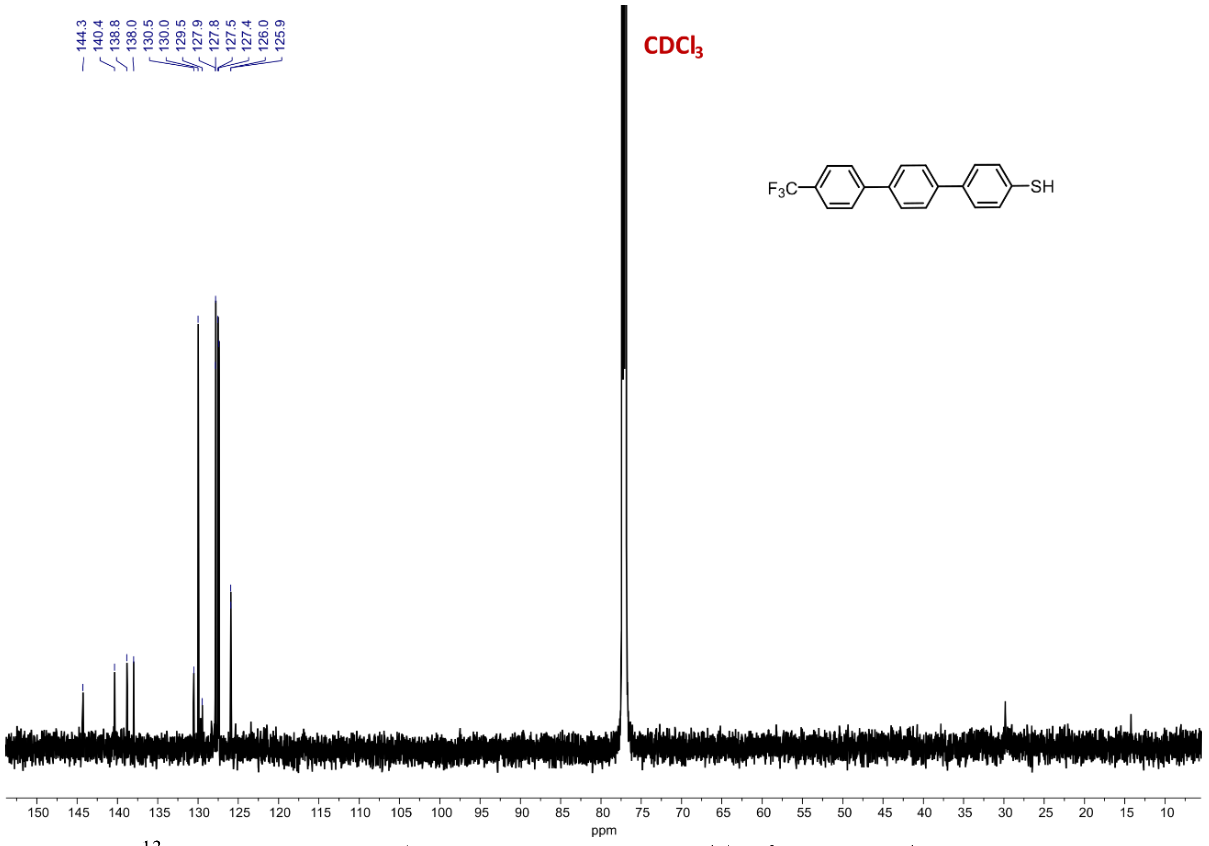

Figure S16. ^{13}C NMR spectrum (151 MHz, 298 K, $CDCl_3$) of compound **(6).**

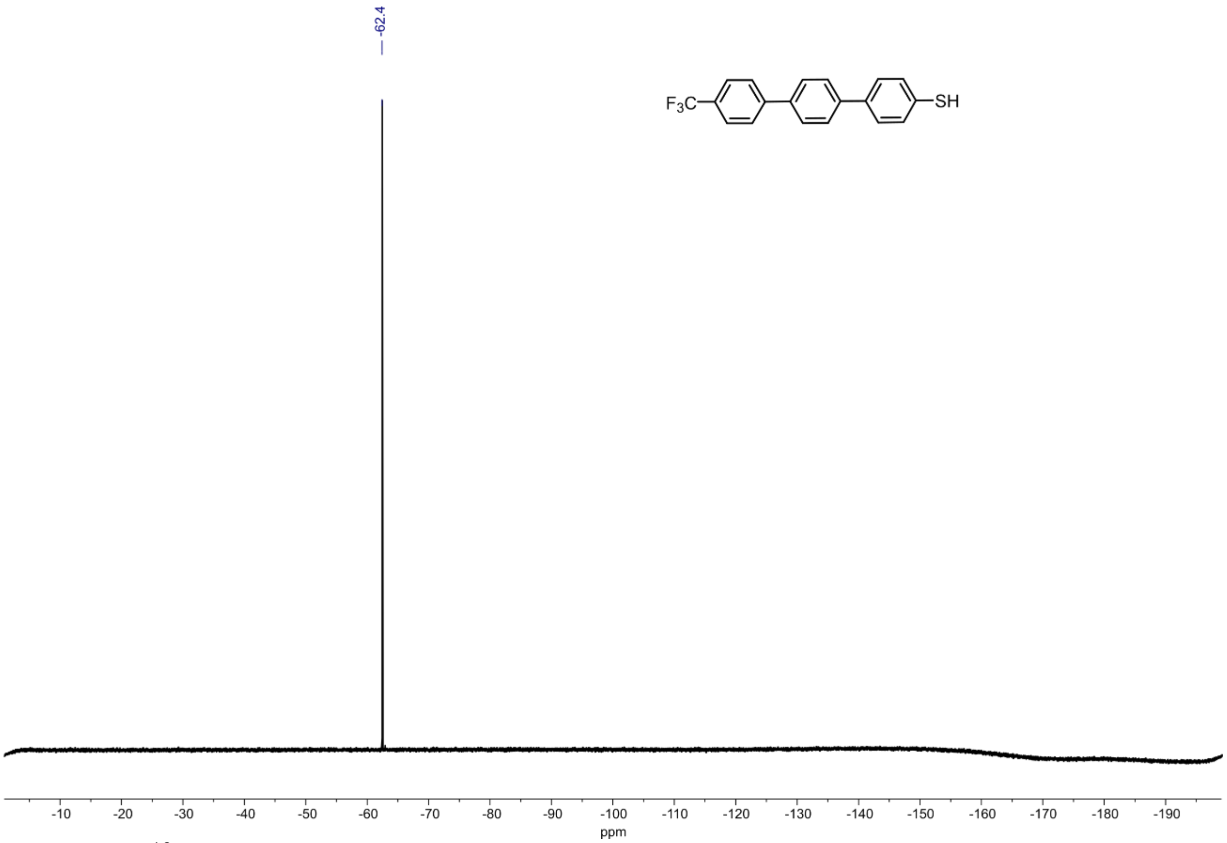

Figure S17. ^{19}F NMR spectrum (376 MHz, 298 K, $CDCl_3$) of compound **(6)**.

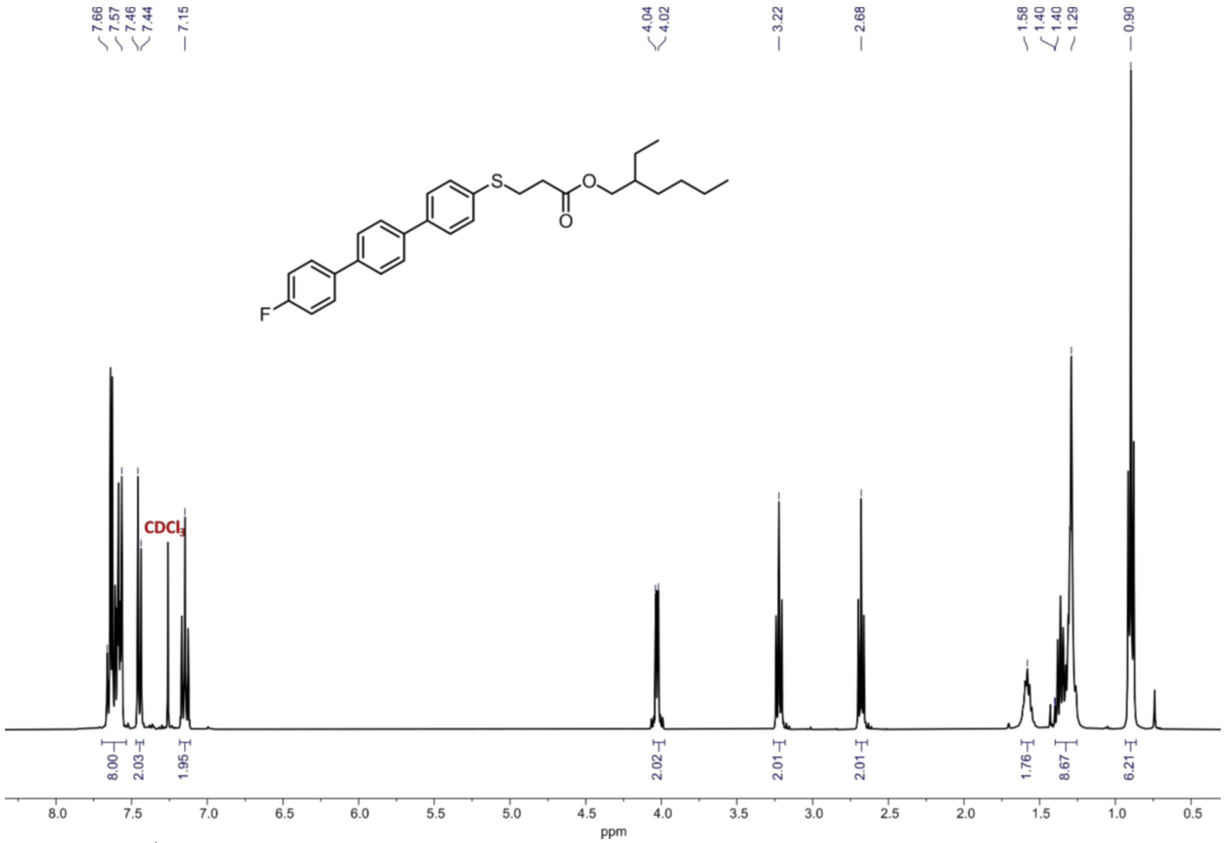

Figure S18. ^{1}H NMR spectrum (400 MHz, 298 K, $CDCl_3$) of compound **(7).**

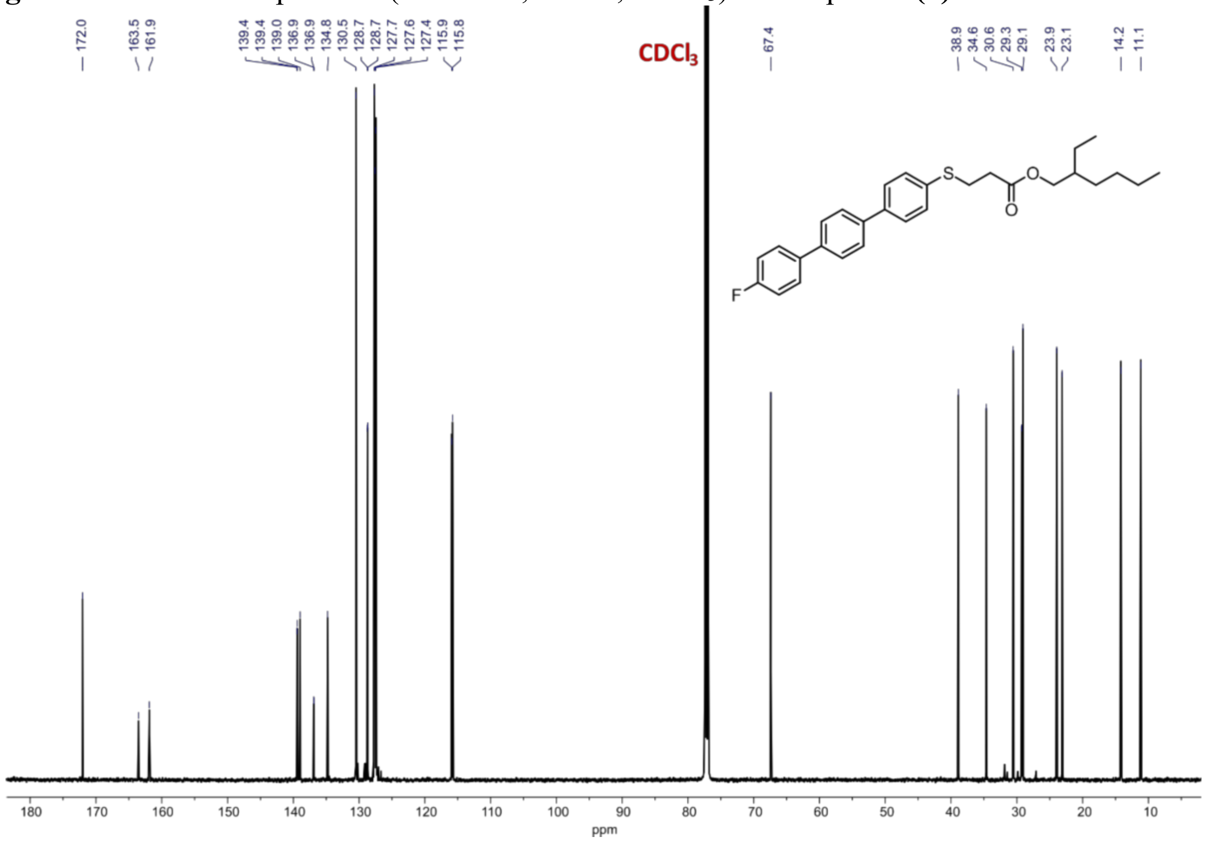

Figure S19. 13C NMR spectrum (151 MHz, 298 K, CDCl3) of compound **(7).**

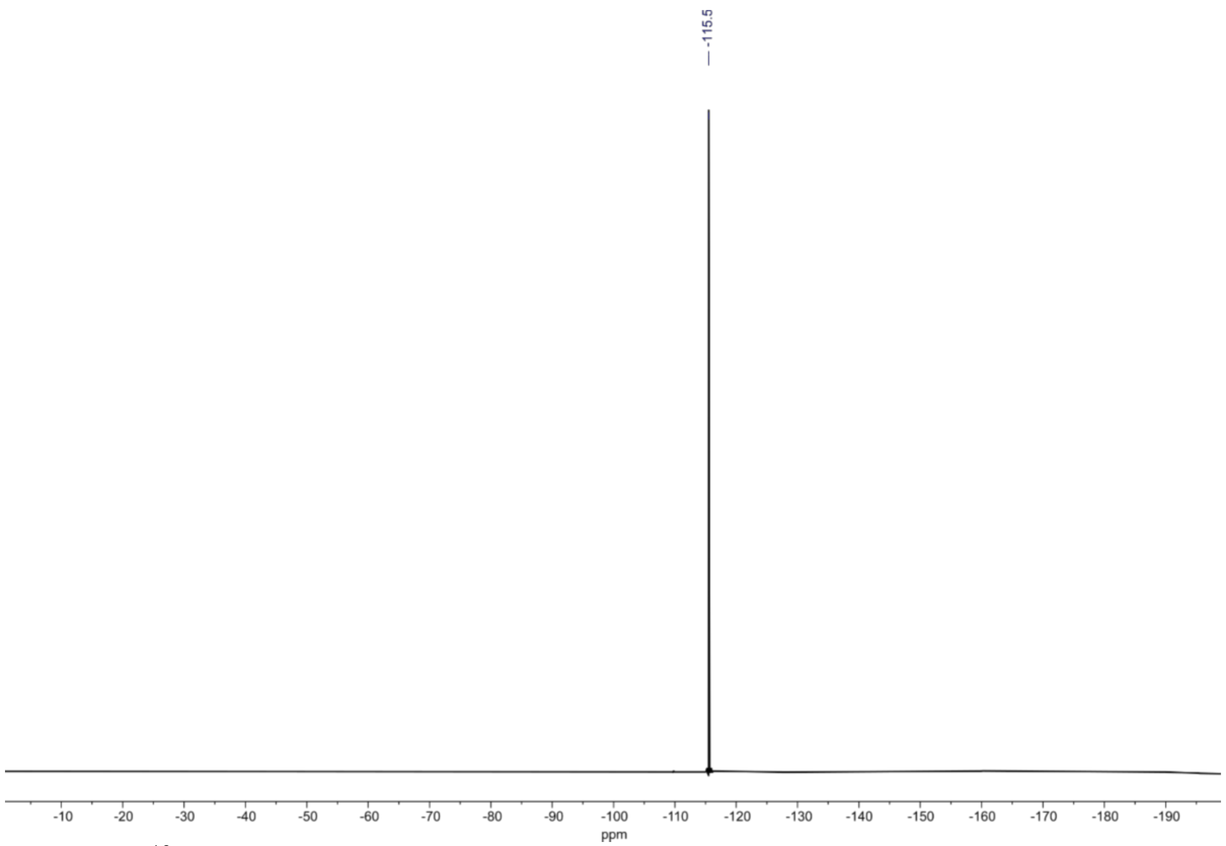

Figure S20. ^{19}F NMR spectrum (376 MHz, 298K, $CDCl_3$) of compound **(7)**.

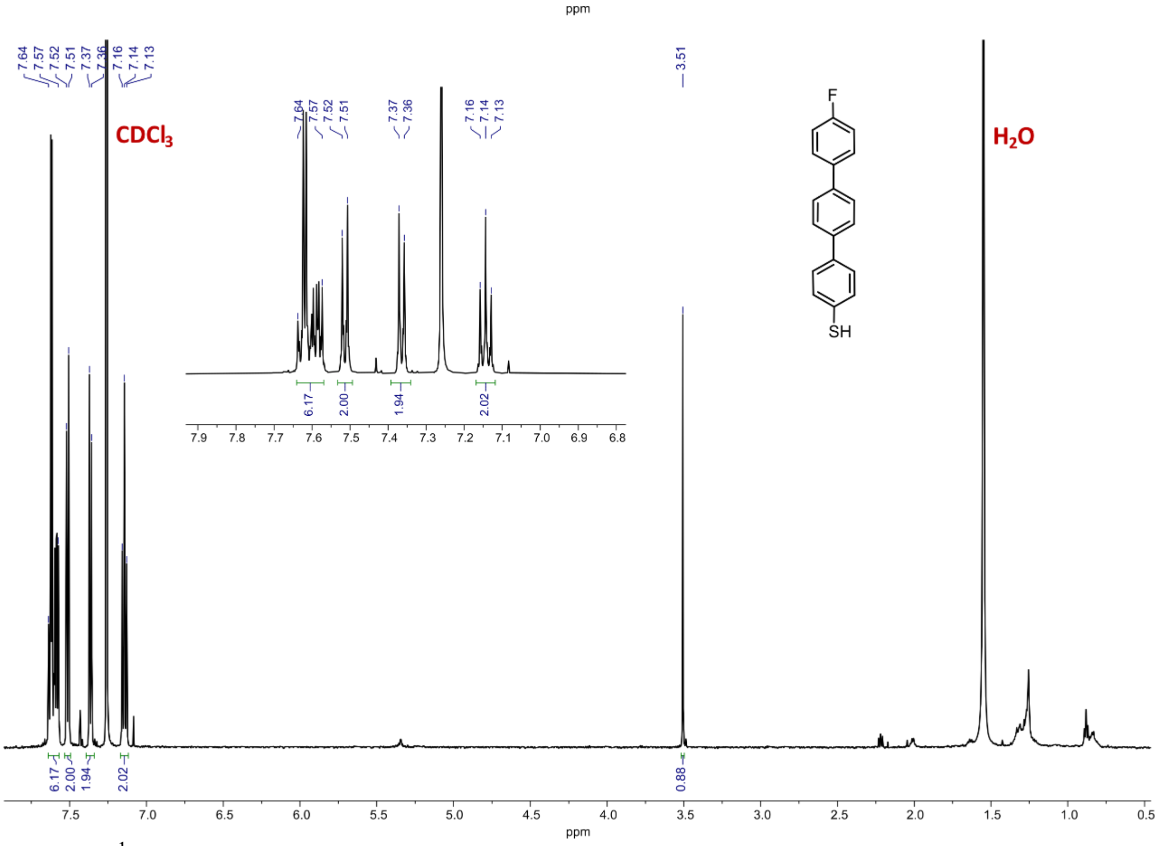

Figure S21. ^{1}H NMR spectrum (600 MHz, 298 K, $CDCl_3$) of compound **(8)**.

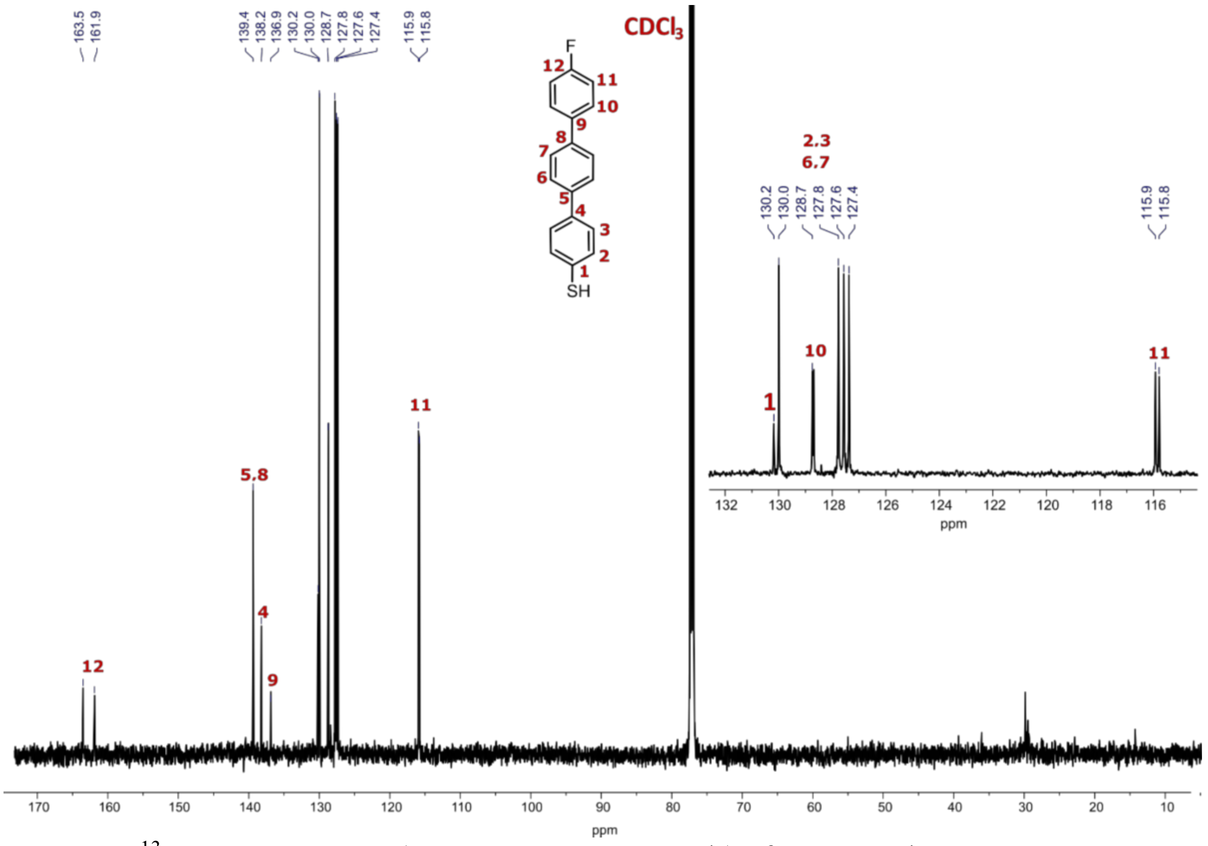

Figure S22. ^{13}C NMR spectrum (151 MHz, 298 K, $CDCl_3$) of compound **(8)**.

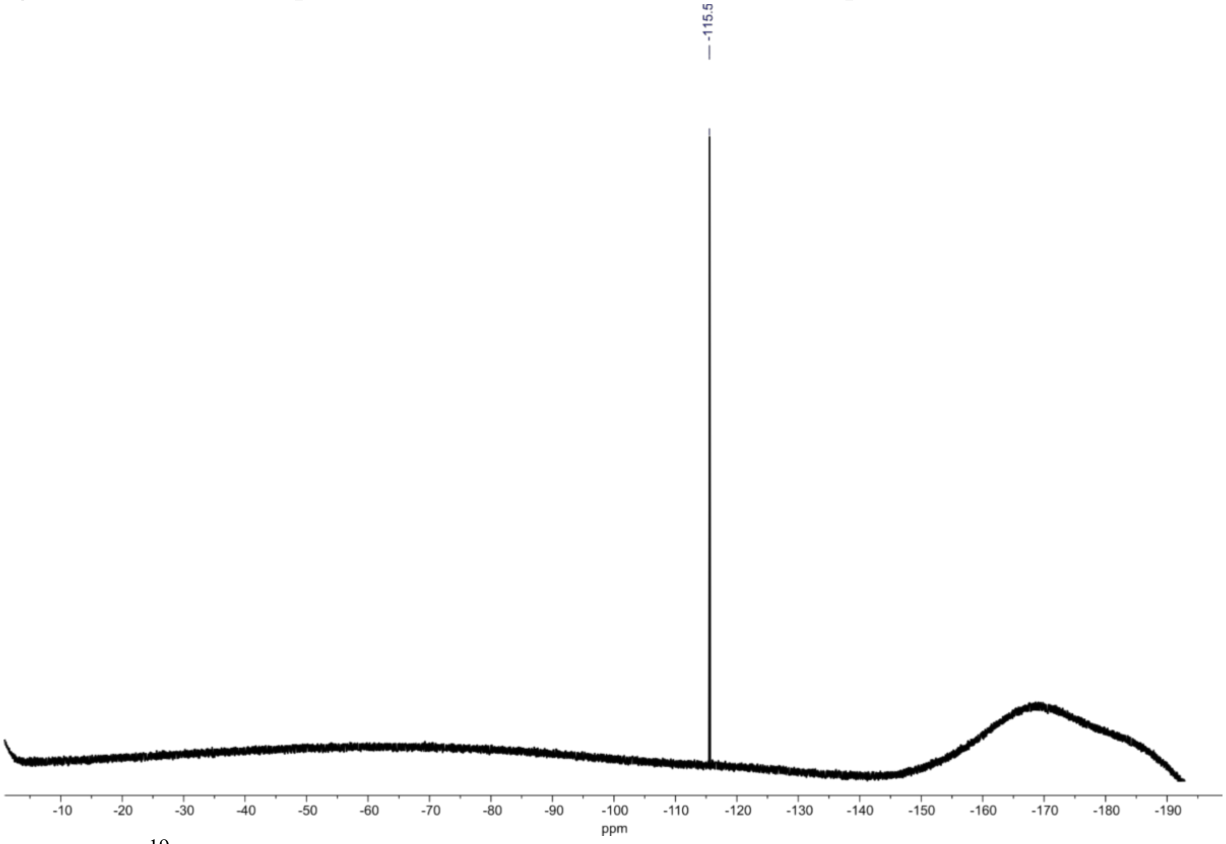

Figure S23. ^{19}F NMR spectrum (376 MHz, 298 K, $CDCl_3$) of compound **(8)**.

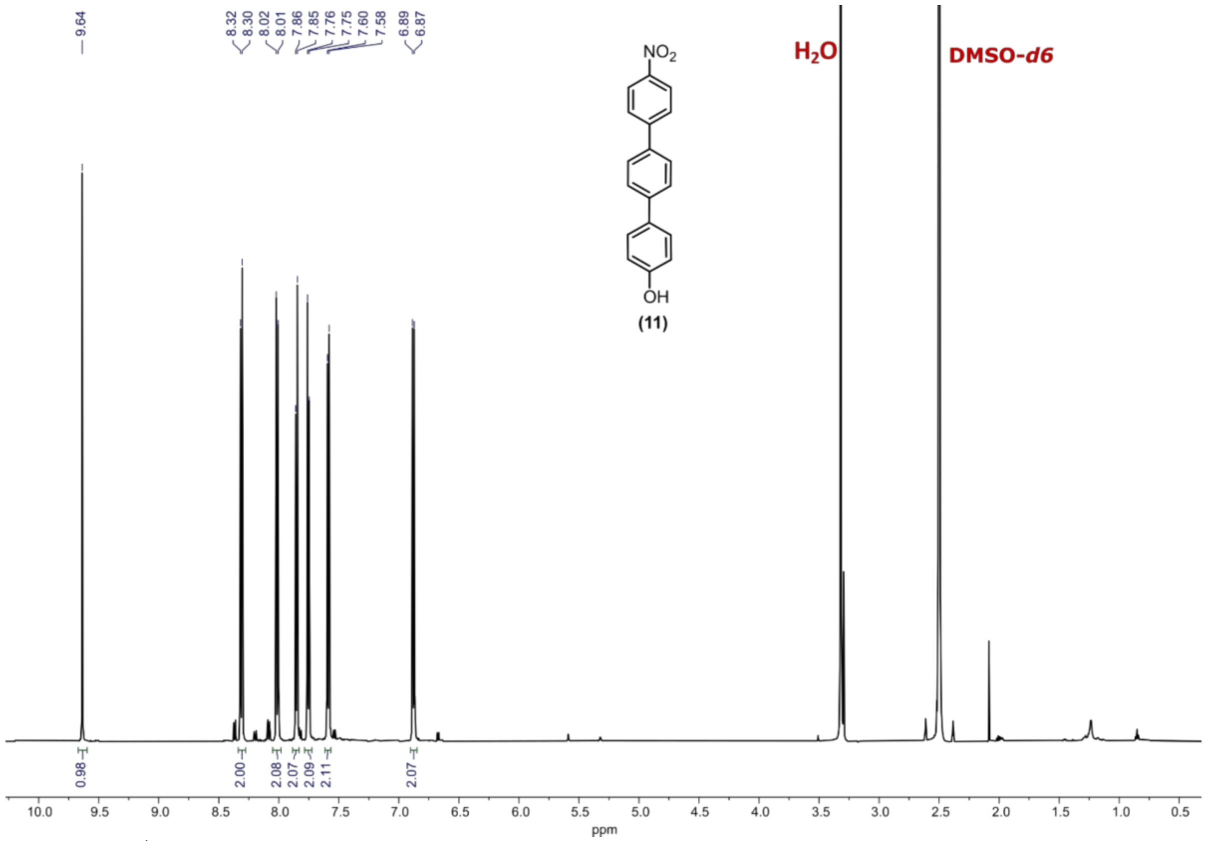

Figure S24. ^{1}H NMR spectrum (600 MHz, 298 K, DMSO-d6) of compound **(9)**.

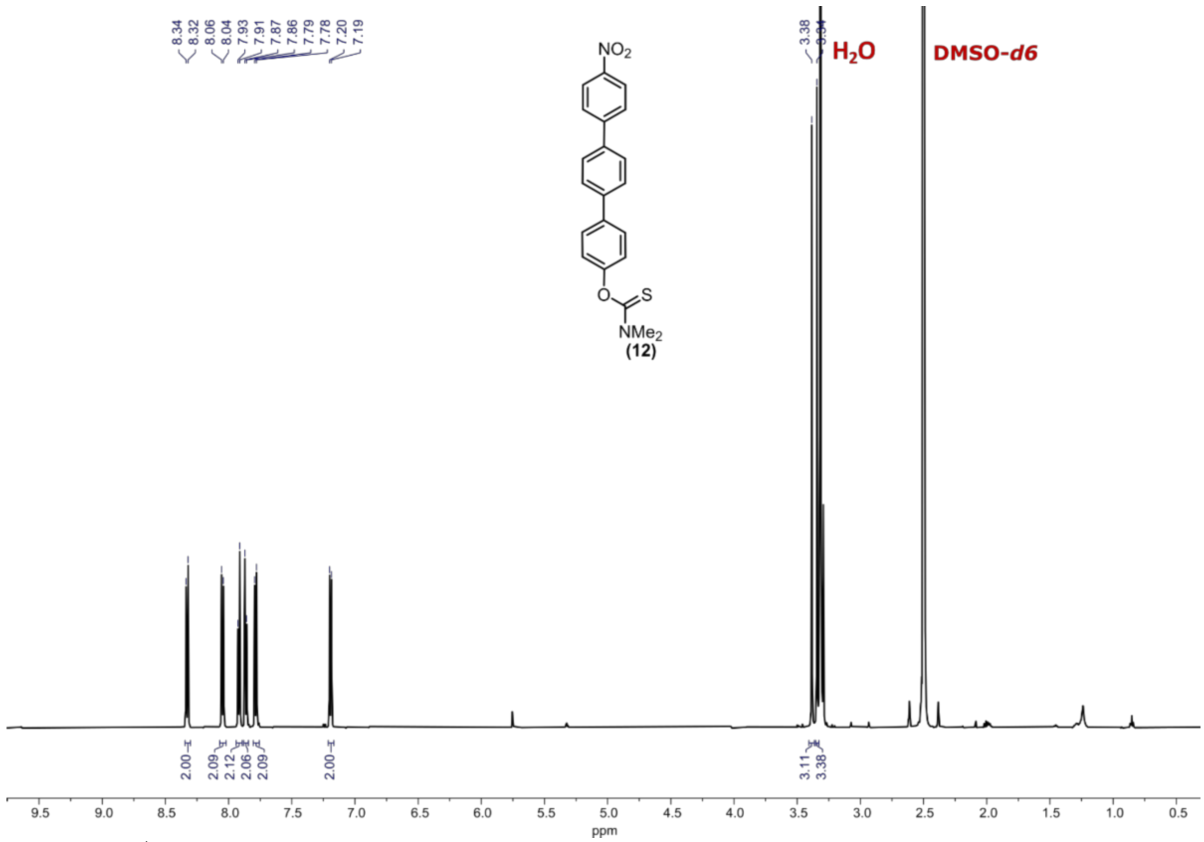

Figure S25. ^{1}H NMR spectrum (600 MHz, 298 K, DMSO-d6) of compound **(10).**

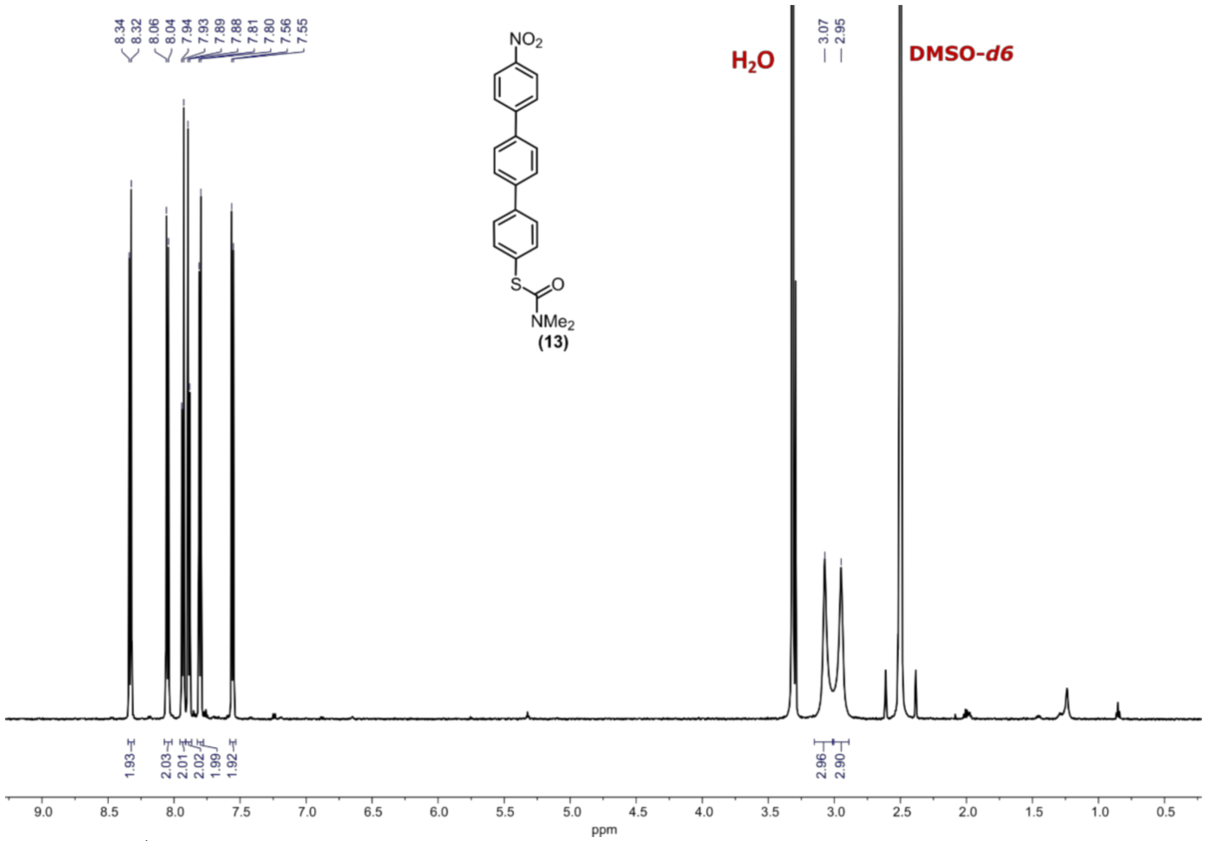

Figure S26. ^{1}H NMR spectrum (600 MHz, 298 K, DMSO-d6) of compound **(11).**

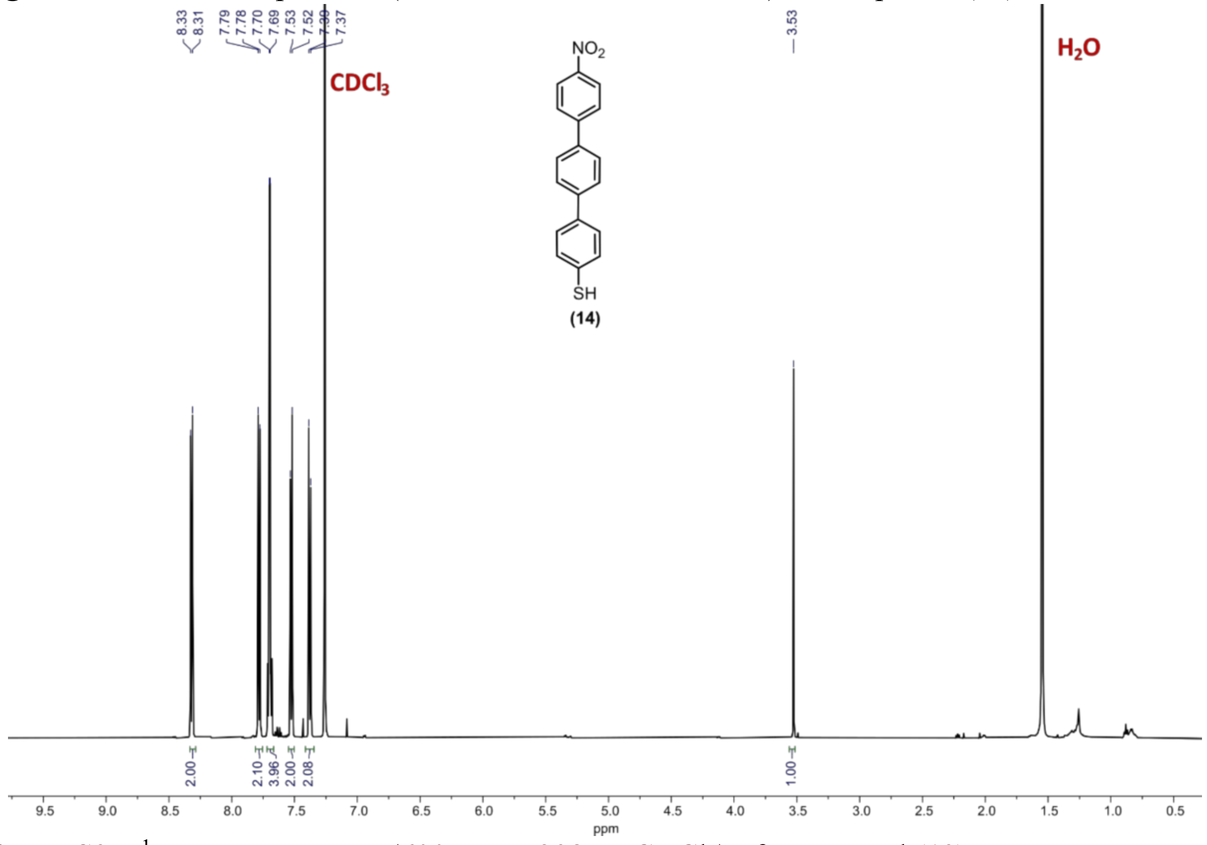

Figure S27. ^{1}H NMR spectrum (600 MHz, 298 K, $CDCl_3$) of compound **(12).**

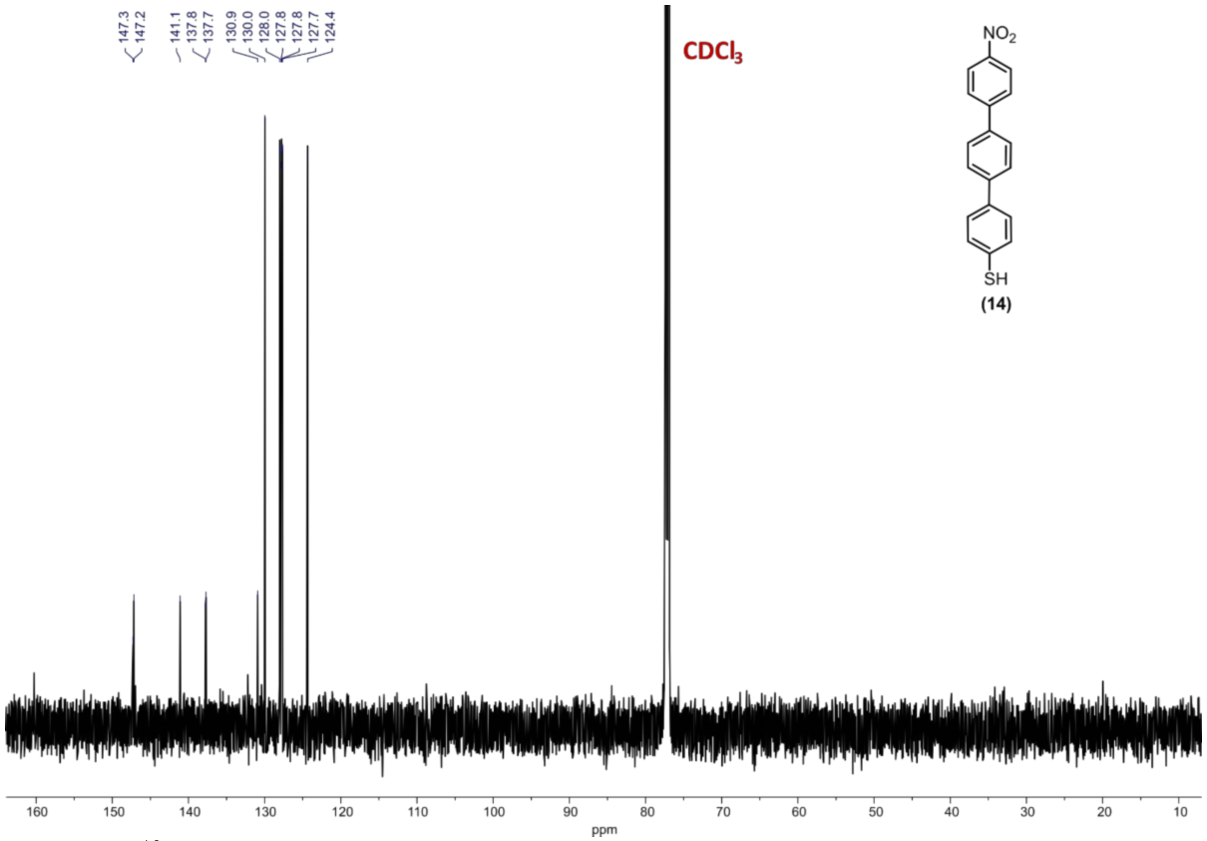

Figure S28. ^{13}C NMR spectrum (151 MHz, 298 K, $CDCl_3$) of compound **(12)**.

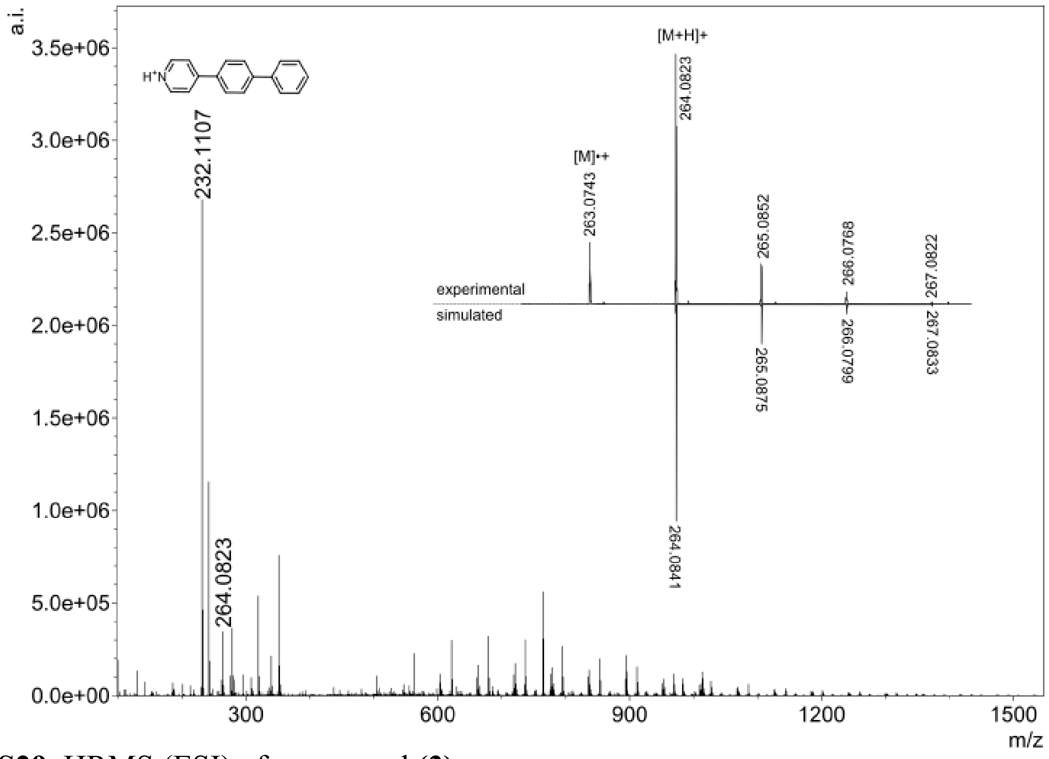

Figure S29. HRMS (ESI) of compound **(3)**.

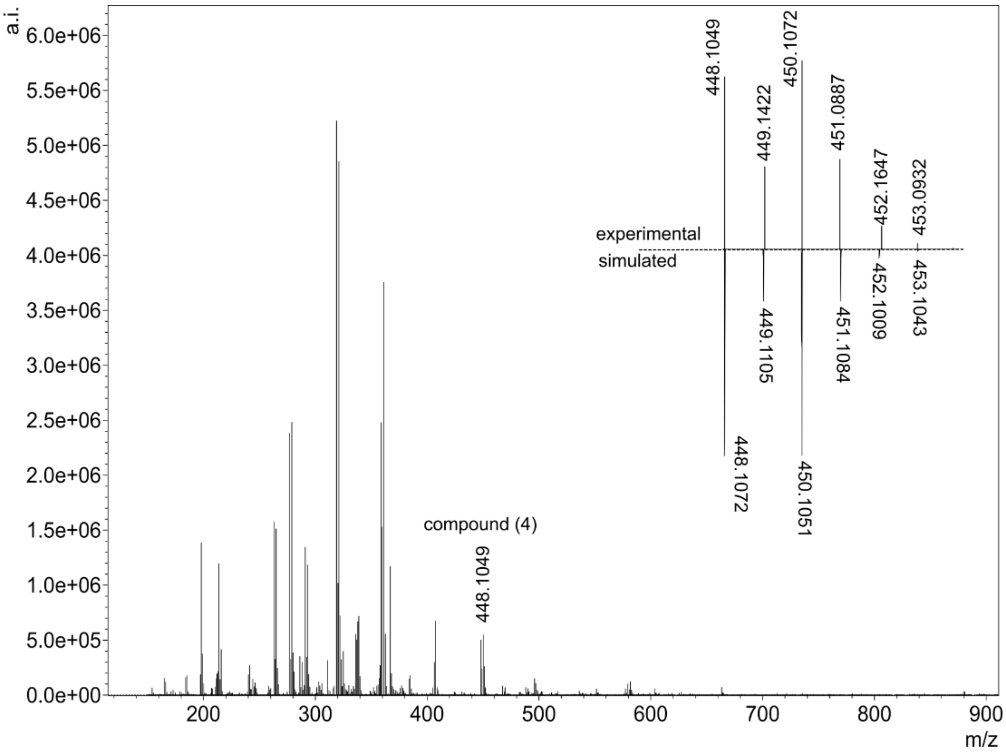

Figure S30. HRMS (APCI) of compound **(4)**.

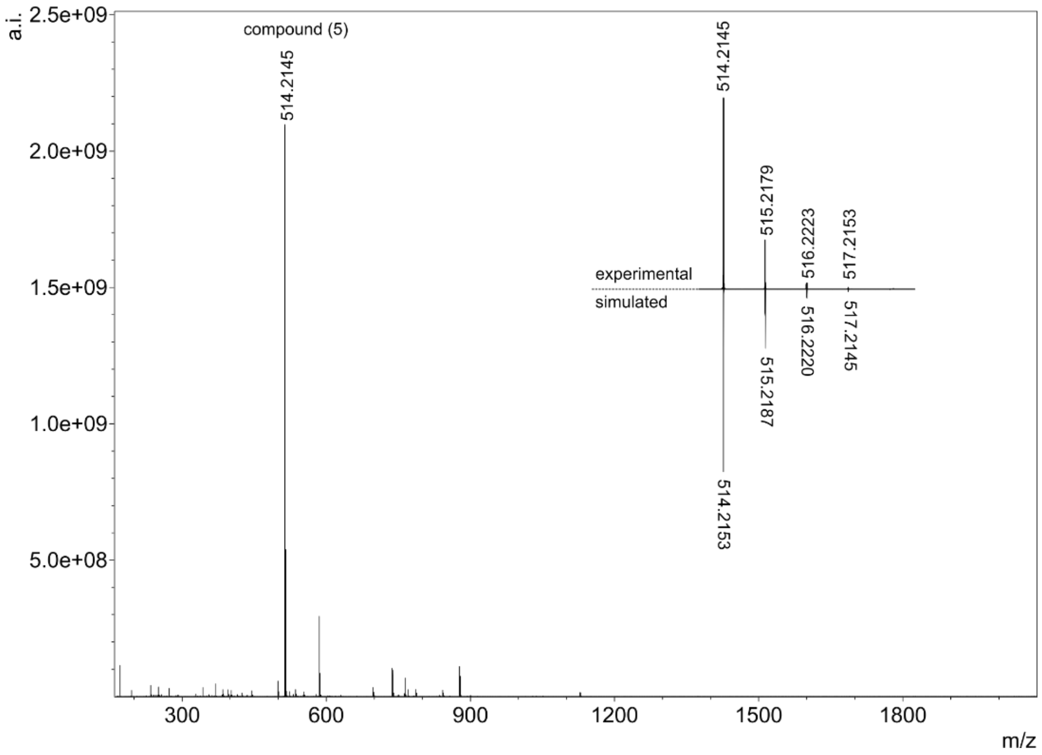

Figure S31. HRMS (MALDI) of compound **(5)**.

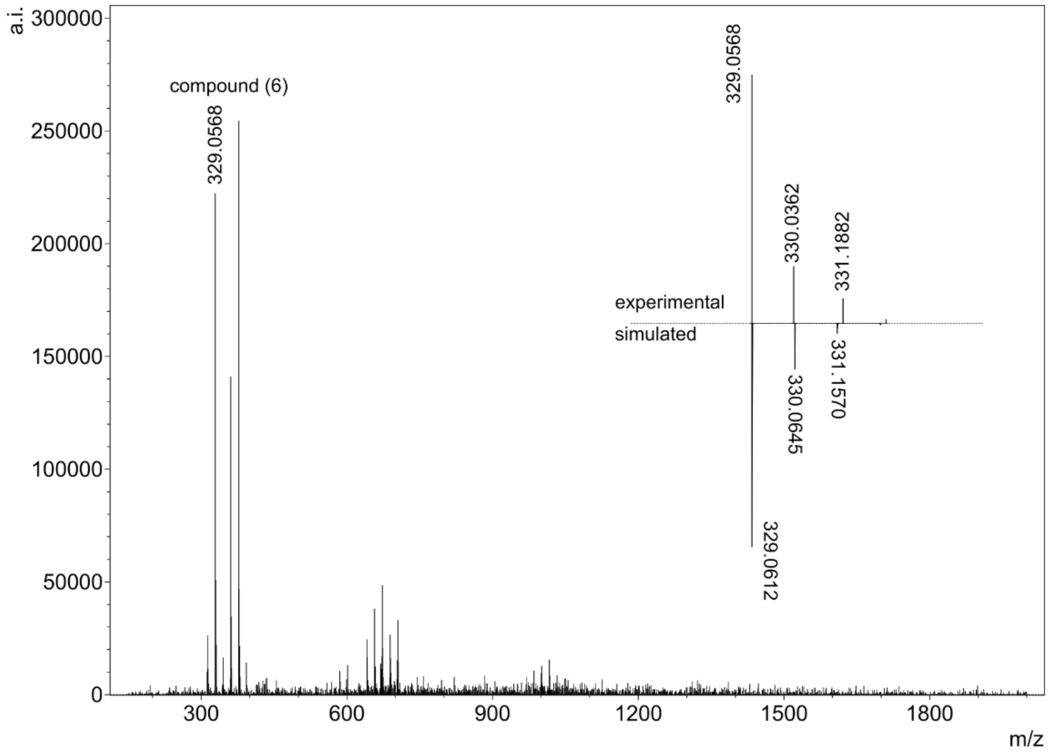

Figure S32. HRMS (APCI) of compound **(6)**.

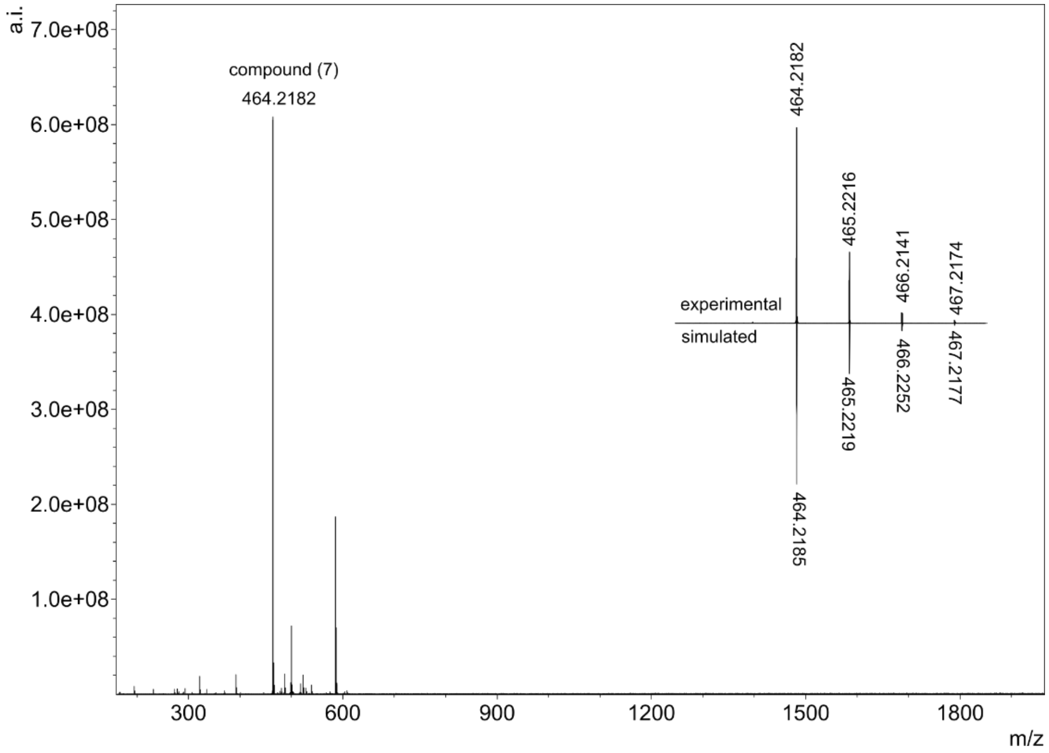

Figure S33. HRMS (MALDI) of compound **(7)**.

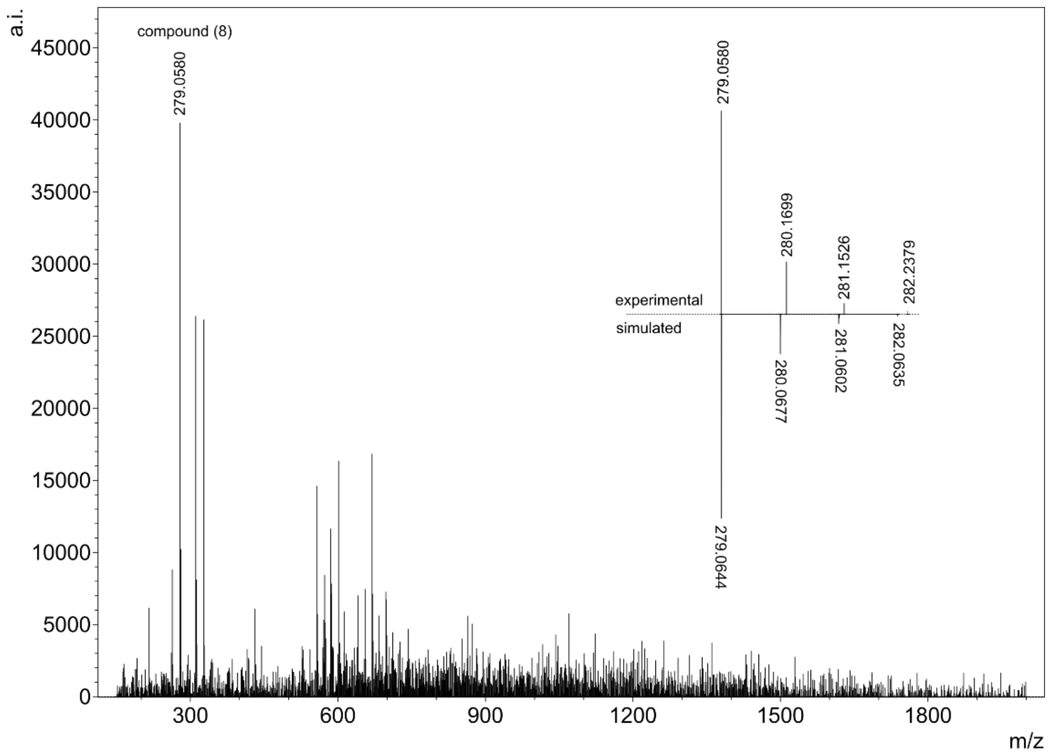

Figure S34. HRMS (APCI) of compound **(8).**

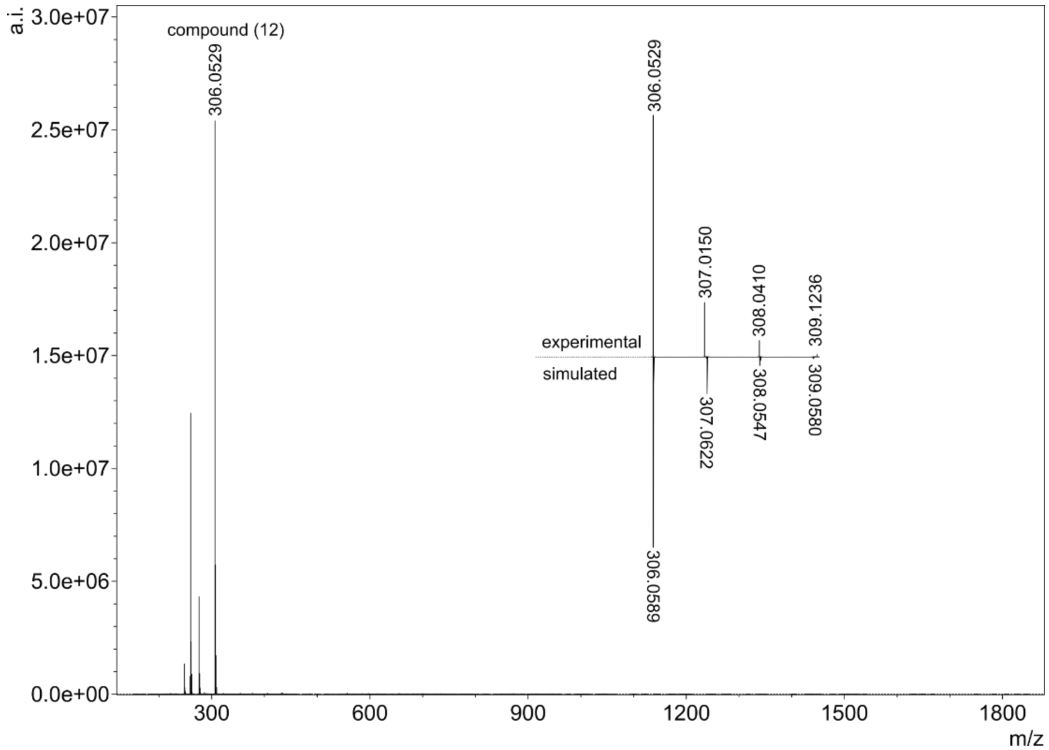

Figure S35. HRMS (APCI) of compound **(12).**

2. XPS Data

Table S 1. Peak positions, FWHM and component percentage of the TPT, FTPT, CF_3TPT, PyBPT and NTPT SAMs on Au/mica.

Peak assignment	Binding Energy / eV	FWHM / eV	%
TPT SAM			
		C 1s	
C-C/C-H	284.1	1.0	75.4
C-S	284.9	1.0	7.9
Shake up	286.7	2.0	6.0
Shake up	290.2	2.3	6.4
Shake up	292.9	2.3	4.4
		S 2p	
Thiolate	161.9	0.8	-
FTPT SAM			
		C 1s	
C-C/C-H	284.1	1.1	76.9
C-S	285.2	1.4	3.7
C-F	286.5	1.0	8.3
Shake up	289.5	2.5	6.4
Shake up	292.4	2.5	4.7
		S 2p	
Thiolate	162.0	0.9	-
		O 1s	
C=O, C-O	531.9	1.8	-
		F 1s	
C-F	686.9	1.2	-
CF_3TPT SAM			
		C 1s	
C-C/C-H	284.1	1.0	72.0
C-S	285.2	1.5	7.8
C-O	287.1	1.5	4.7
Shake up	290.1	2.5	6.0
$-CF_3$	291.8	0.6	5.3
Shake up	293.0	2.5	4.3
		S 2p	
Thiolate	161.9	0.8	-
		O 1s	
C=O, C-O	532.8	1.6	-
		F 1s	
$-CF_3$	687.3	1.6	-

PyBPT SAM			
		C 1s	
C-C/C-H	284.3	1.1	57.4
C-S/C-N	285.3	1.2	24.6
C-O/Shake up	286.9	1.5	8.0
Shake up	289.3	2.2	4.9
Shake up	292.2	2.5	5.1
		S 2p	
Thiolate	162.0	0.7	-
		N 1s	
N_{Py}	398.4	0.9	60.0
N_{Py} with H modified	399.8	1.6	40.0
		O 1s	
C=O, C-O	532.5	1.9	-
NTPT SAM			
		C 1s	
C-C/C-H	284.3	1.1	65.12
C-S/C-N	285.2	1.5	19.1
C-O	287.1	1.5	5.2
Shake up	289.9	2.5	6.2
Shake up	293.0	2.5	4.4
		S 2p	
Thiolate	161.9	0.7	-
		N 1s	
$-NO_2$	405.6	1.4	-
		O 1s	
$-NO_2$	532.4	1.4	83.6
Adsorbed H_2O	534.1	1.5	16.4

3. STM and LEED Data Analysis

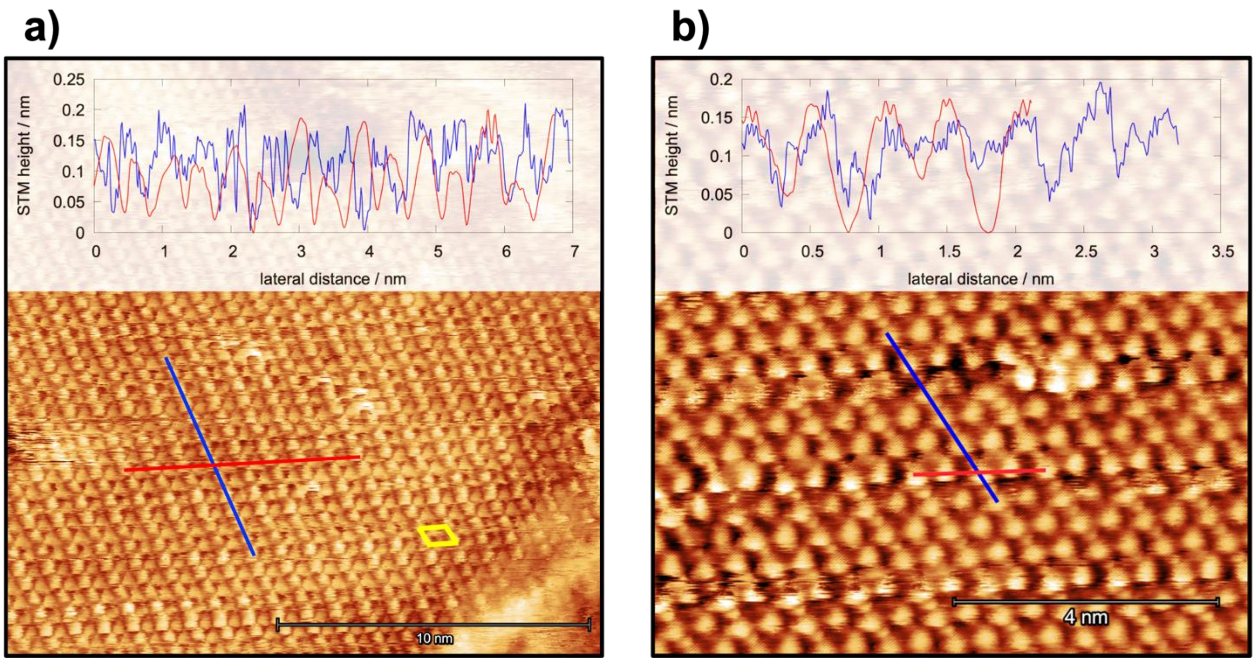

Figure S36. STM data of the TPT SAM on Au(111). Both shown images are drift-corrected (see Experimental for details). a) A highly ordered structure is observed for a large scan area (19×19 nm^2) with line profile analysis. The hexagonal unit cell of the $2\sqrt{3} \times \sqrt{3}$ R30° with respect to the Au substrate is highlighted in yellow. b) Line profile analysis. Both images were acquired under identical STM imaging conditions: -0.7 V, 0.4 nA, 293 K.

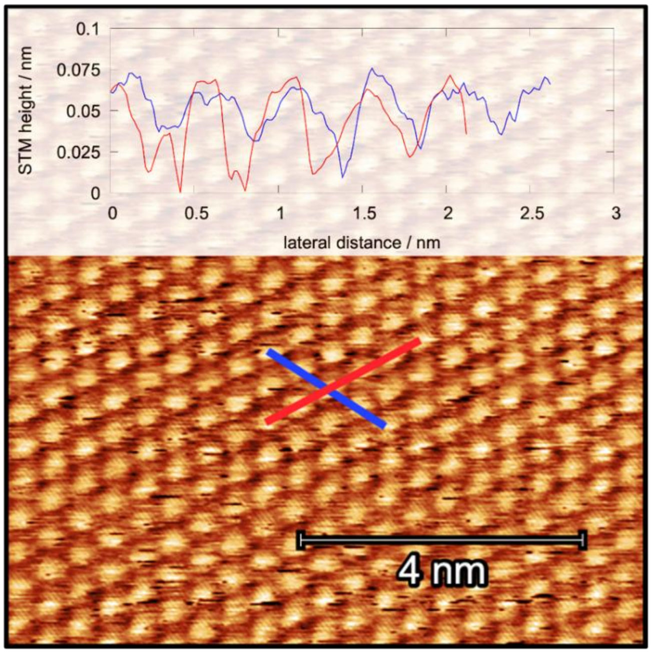

Figure S37. Line profile analysis of the FTPT SAM on Au(111). STM conditions: -0.1 V, 1 nA, 293 K.

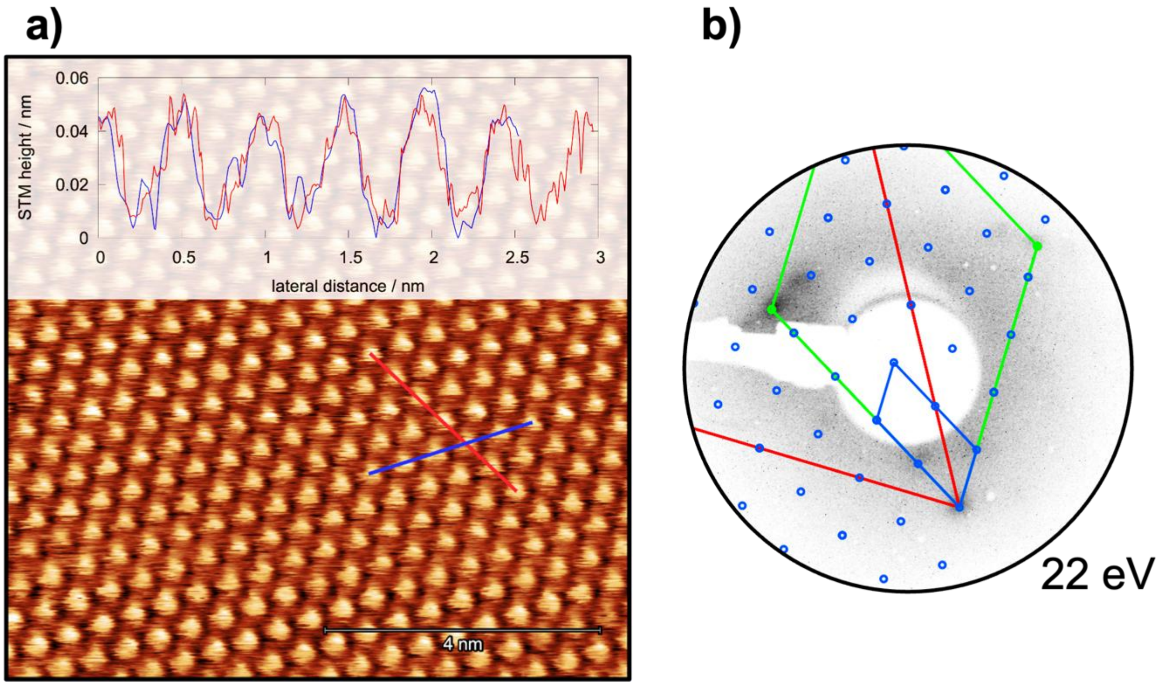

Figure S38. STM and LEED data of the CF_3TPT SAM on Au(111). a) Line profile analysis, STM conditions: -0.1 V, 1 nA, 293 K. b) Analysis of the LEED pattern obtained at an electron beam energy of 22 eV revealing $\sqrt{3} \times \sqrt{3}$ R30° superstructure (green). Some additional diffraction spots most probably result from simultaneous presence of the $2\sqrt{3} \times 4\sqrt{3}$ R30° superstructure (blue) in this sample.

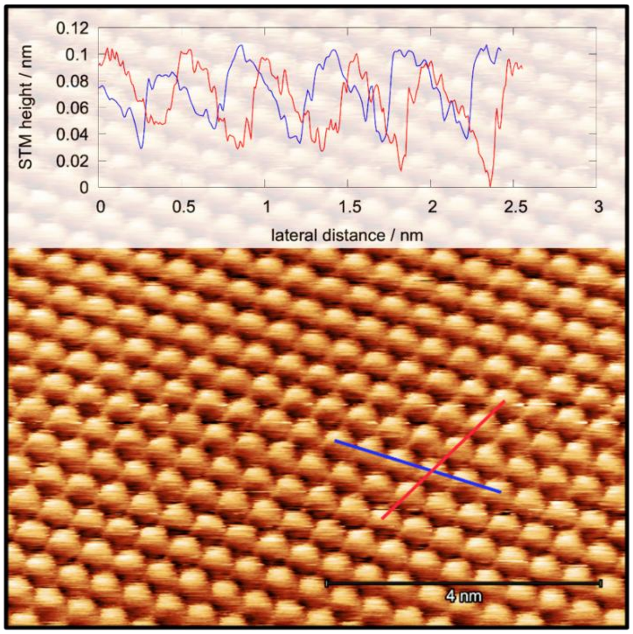

Figure S39. Line profile analysis of the PyBPT SAM on Au(111). STM conditions: -0.1 V, 1 nA, 293 K.

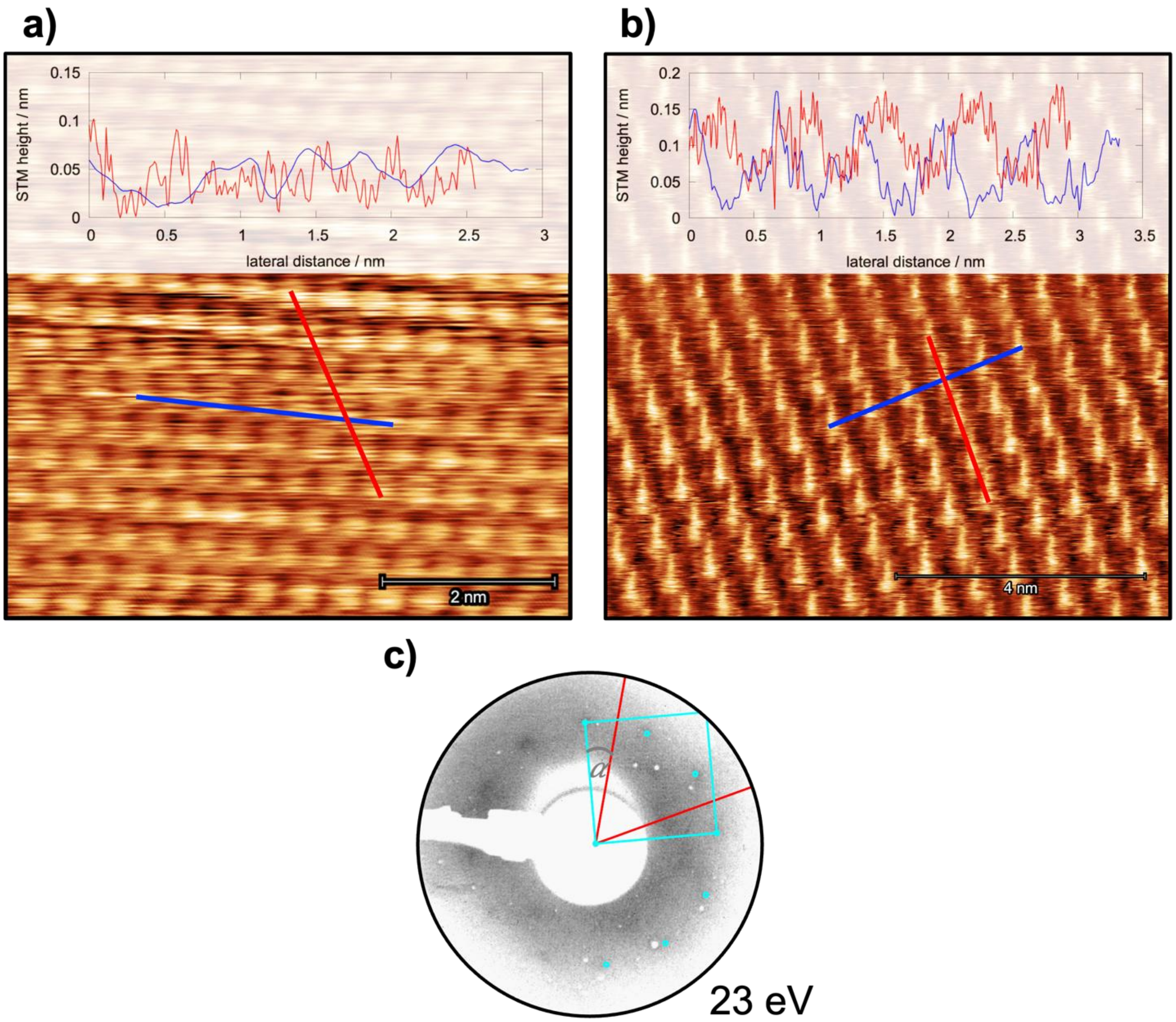

Figure S40. STM images a), b) and LEED pattern c) of the NTPT SAM on Au(111). a) Line profile analysis. A densely packed hexagonal $\sqrt{3} \times \sqrt{3}$ R30° superstructure with respect to the Au substrate is observed. b) Line profile analysis of a squared superstructure. STM conditions: -0.1 V, 1 nA, 293 K. c) LEED pattern obtained at an electron beam energy of 23 eV. The squared unit cell is highlighted in light blue. The hexagonal unit cell of the Au substrate is marked with red. The rotation angle α between the first lattice vector $\overrightarrow{a_1}$ and the first substrate vector is highlighted in grey.

References

(1) Bartucci, M. A.; Wierzbicki, P. M.; Gwengo, C.; Shajan, S.; Hussain, S. H.; Ciszek, J. W. Synthesis of dihydroindolizines for potential photoinduced work function alteration. *Tetrahedron Lett.* **2010**, *51*, 6839-6842.

(2) Ozores, H. L.; Amorín, M.; Granja, J. R. Self-Assembling Molecular Capsules Based on α,γ-Cyclic Peptides. *J. Am. Chem. Soc.* **2017**, *139*, 776−784.

(3) Wang, Y.; Frattarelli, D. L.; Facchetti, A.; Cariati, E.; Tordin, E.; Ugo, R.; Zuccaccia, C.; Macchioni, A.; Wegener, S. L.; Stern, C. L.; Ratner, M. A.; Marks, T. J. Twisted π-Electron System Electrooptic Chromophores. Structural and Electronic Consequences of Relaxing Twist-Inducing Nonbonded Repulsions. *J. Phys. Chem. C* **2008,** *112,* 8005–8015.

(4) Itoh, T.; Mase, T. A General Palladium-Catalyzed Coupling of Aryl Bromides/Triflates and Thiols. *Org. Lett.* **2004***, 6,* 4587–4590.

(5) Waske, P.; Wächter, T.; Terfort, A.; Zharnikov, M. Nitro-Substituted Aromatic Thiolate Self-Assembled Monolayers: Structural Properties and Electron Transfer upon Resonant Excitation of the Tail Group. *J. Phys. Chem. C* **2014**, *118*, 26049−26060.
